# Prevalence, Evolution, and *cis*-Regulation of Diel Transcription in *Chlamydomonas reinhardtii*

**DOI:** 10.1534/g3.114.015032

**Published:** 2014-10-28

**Authors:** Nicholas Panchy, Guangxi Wu, Linsey Newton, Chia-Hong Tsai, Jin Chen, Christoph Benning, Eva M. Farré, Shin-Han Shiu

**Affiliations:** * Genetics Program, Michigan State University, East Lansing, Michigan 48824; † Cellular and Molecular Biology Program, Michigan State University, East Lansing, Michigan 48824; ‡ Department of Plant Biology, Michigan State University, East Lansing, Michigan 48824; § MSU-DOE Plant Research Laboratory, Michigan State University, East Lansing, Michigan 48824; ** Department of Computer Science & Engineering, Michigan State University, East Lansing, Michigan 48824; †† Department of Biochemistry and Molecular Biology, Michigan State University, East Lansing, Michigan 48824

**Keywords:** green algae, diel expression, transcriptomics, evolution, gene regulation, *cis*-regulatory element

## Abstract

Endogenous (circadian) and exogenous (*e.g.*, diel) biological rhythms are a prominent feature of many living systems. In green algal species, knowledge of the extent of diel rhythmicity of genome-wide gene expression, its evolution, and its *cis*-regulatory mechanism is limited. In this study, we identified cyclically expressed genes under diel conditions in *Chlamydomonas reinhardtii* and found that ~50% of the 17,114 annotated genes exhibited cyclic expression. These cyclic expression patterns indicate a clear succession of biological processes during the course of a day. Among 237 functional categories enriched in cyclically expressed genes, >90% were phase-specific, including photosynthesis, cell division, and motility-related processes. By contrasting cyclic expression between *C. reinhardtii* and *Arabidopsis thaliana* putative orthologs, we found significant but weak conservation in cyclic gene expression patterns. On the other hand, within *C. reinhardtii* cyclic expression was preferentially maintained between duplicates, and the evolution of phase between paralogs is limited to relatively minor time shifts. Finally, to better understand the *cis* regulatory basis of diel expression, putative *cis*-regulatory elements were identified that could predict the expression phase of a subset of the cyclic transcriptome. Our findings demonstrate both the prevalence of cycling genes as well as the complex regulatory circuitry required to control cyclic expression in a green algal model, highlighting the need to consider diel expression in studying algal molecular networks and in future biotechnological applications.

Diel (24-hr, day/night periods) cycles dictate physiological changes at different times of day in many organisms. The timing of these physiological oscillations is regulated by a combination of environmental, metabolic, and circadian signaling processes (Farre 2012; Kinmonth-Schultz *et al.* 2013; Song *et al.* 2013; Fonken and Nelson 2014). For example, circadian clock mutants lead to phase changes under entrained diel conditions (*i.e.*, light-dark cycles) and changes in photoperiod sensitivity (Yanovsky and Kay 2002; McNabb and Truman 2008). Oscillations can be a direct adaptation to environmental cycles, for example, restricting photosynthesis and protection against ultraviolet radiation to periods of light. Diel cycles also influence biotic responses such as defense mechanisms (Arimura *et al.* 2008; Goodspeed *et al.* 2012; Baldwin and Meldau 2013) and mutualistic interactions (Frund *et al.* 2011; Lehmann *et al.* 2011). Mechanistically, many of these cycling responses are regulated at the transcriptional level. For example, in the green alga *Chlamydomonas reinhardtii*, oscillations in starch levels are partially regulated by the cyclic expression of adenosine diphosphate-glucose pyrophosphorylase (Ral *et al.* 2006). However, some circadian regulated processes are controlled at the posttranscriptional level (Kojima *et al.* 2011) and/or by the interaction between transcriptional and posttranslational regulation (Kinmonth-Schultz *et al.* 2013; Song *et al.* 2013). Early transcriptome analyses of three model organisms, *Arabidopsis thaliana*, *Drosophila melanogaster*, and *Mus musculus*, indicated that between 1 and 10% of genes exhibit circadian oscillation with periods of ~24 hr (Doherty and Kay 2010). Moreover, in photosynthetic organisms, 30–90% of genes cycle under diel conditions (Michael *et al.* 2008; Monnier *et al.* 2010; Shi *et al.* 2010; Filichkin *et al.* 2011). In land plants, approximately a third of the genes that cycle in light/dark are also circadian regulated (Michael *et al.* 2008; Filichkin *et al.* 2011). Several *cis*-regulatory elements (CREs) necessary for circadian-regulated gene expression have been identified (Michael and McClung 2002; Harmer and Kay 2005; Michael *et al.* 2008), although it remains an open question how well the identified CREs explain global cyclic expression patterns.

The green alga *C. reinhardtii* has been used extensively to study physiological processes under the control of circadian and/or diel cycle (Mittag *et al.* 2005; Matsuo and Ishiura 2010). *C. reinhardtii*’s size, short life-cycle, and extensive genetic tool set make it an ideal model organism (Harris 2001), particularly for studies such as experimental evolution from single to multicellularity (Ratcliff *et al.* 2013) and the genetic engineering of triacylglycerol accumulation in algae (Grossman *et al.* 2007; Hu *et al.* 2008; Siaut *et al.* 2011). *C. reinhardtii* also has been used to study rhythmic responses to light (Bruce 1970), ammonium (Byrne *et al.* 1992), and nitrogen availability (Pajuelo *et al.* 1995). However, studies of cyclic expression in *C. reinhardtii* have been limited to single (Mittag *et al.* 2005; Matsuo and Ishiura 2010) or relatively small sets of genes (Kucho *et al.* 2005). Despite the large evolutionary distance, there are some conserved elements between both the circadian (Corellou *et al.* 2009; Matsuo and Ishiura 2010) and the photoperiodic (Romero and Valverde 2009) oscillators of flowering plants and green algae, raising the question whether and to what extent cyclic expression is conserved. Therefore, a genome-wide analysis of cyclic expression in *C. reinhardtii* can provide insight not only into cyclic physiological behavior in green algae but also how this behavior has evolved in divergent lineages of the *Plantae*. Such an analysis will also be relevant to economically important processes in algae such as oil production.

In this study, we examined gene expression patterns under diel conditions in *C. reinhardtii*. We characterized the prevalence of cycling gene expression in the *C. reinhardtii* genome and observed that genes involved in distinct biological processes are consistently expressed at certain times during the day/night cycle. We also investigated the conservation of cyclic expression patterns between orthologs in *C. reinhardtii* and *A. thaliana*, which diverged ~650−800 million years ago (Sanderson *et al.* 2004) and the evolution of cycling paralogous genes. Finally, to understand the *cis*-regulatory basis of diel expression, we identified putative CREs (pCREs) associated with cyclic expression at different phases and investigated how these pCREs can be used to predict cycling gene expression.

## Material and Methods

### Growth of *Chlamydomonas reinhardtii* cultures

*C. reinhardtii* dw15.1 was grown in Tris-acetate-phosphate media in flasks without aeration, shaken at 100 rpm, at 22°. Although the acetate present in this media provides an alternative source of carbon, allowing for *C. reinhardtii* to grow in the dark, previous studies have shown that that the cell-cycle (Voigt and Munzner 1987; Davies and Grossman 1994) and other metabolic cycles (Ral *et al.* 2006) are still synchronized in *C. reinhardtii* grown in acetate-containing media under light/dark cycles. Additionally, the amplitude and phase of cell-cycle gene expression in our study and in previous studies where cultures were grown under autotrophic conditions (Bisova *et al.* 2005) are similar (Supporting Information, Figure S1). An initial 200-mL culture was grown to a density of 25 million cells mL^−1^ in constant light (50 μmol s^−1^ m^−2^) and used to set up 50 mL cultures of 0.5 million cells mL^−1^ that were transferred to 12-hr light (50 μmol s^−1^ m^-2^) and 12-hr dark conditions for 48 hr before sampling. Two biological replicates were collected every 3 hr between ZT (*i.e.*, Zeitgeber time, hours since last dawn) 0 and ZT 21. Each sample originated from an independent 50-mL culture. Samples collected during the light to dark or dark to light transition were taken just before the change of conditions. For collection, 2 mL of the culture was placed in a 2-mL tube and centrifuged at max speed in at 4° for 10 min. Amber tubes were used for samples collected during the dark period and the supernatant was removed under weak green light. The pellets were snap frozen in liquid nitrogen. The frozen samples were ground using the QIAGEN tissue lyser for RNA extraction.

### RNA sequencing

RNA was extracted using the Omega eZNA Plant RNA kit. The RNA was eluted in 50 μL of diethylpyrocarbonate-H_2_O, and the concentration was measured using a Nanodrop (Thermo-Fisher). A portion of the RNA was diluted to 1 ng μL^−1^ to check the RNA Integrity Number with a Bioanalyzer (Agilent). All samples had a RNA Integrity Number equal to or greater than 7. Library preparation and sequencing was performed at the MSU-Research Technology Support Facility using the Illumina Tru-Seq Stranded kit with an Illumina HiSequation 2500. Eight samples were sequenced in each lane using a custom bar-coding, but the two biological replicates from the same time point were run in separate lanes. The average number of RNA-Seq reads per sample was 1.81e7, and they ranged between 7.07e6 and 2.58e7. The reads from each of 16 samples (eight time points, two samples each time point) were mapped to the *C. reinhardtii* genome (version 4.3 from Phytozome) using Tophat (Trapnell *et al.* 2009) with default parameters except for intron length (min 13, max 8712) and max-multi-hits (1). Gene models on nonchromosomal fragments were not considered. Fragments per kilobase of transcript per million mapped reads (FPKM) per gene were calculated using Cufflinks (Trapnell *et al.* 2010) with parameter –I 8712. A high percentage of reads mapped to the genome: the least mapped sample had 82% of reads mapped and the average of all samples was 85%. Upper quartile normalization was applied to all samples to correct for technical variation as recommend in Bullard *et al.* (2010). The two biological replicates were appended and used as two consecutive days for downstream analysis. Raw read data are available through the National Center for Biotechnology Information Sequence Read Archive, BioProject accession [PRJNA264777].

### Identification of cycling genes

Two programs were used to identify cyclic patterns of expression in FPKM data, COSPOT (which is described in Panda *et al.* 2002) and an application of the discrete Fourier transform (DFT). The DFT has been applied previously to the analysis of cyclic expression using RNA-Seq data (Rodriguez *et al.* 2013), but our method is based primarily on PRIISM (Rosa *et al.* 2012). We chose to use both COSPOT and the DFT in conjunction because we found that the combination of methods had superior coverage of known cycling genes without a substantial increase in the expected false positive rate (see File S1)

In our application, we take the DFT of each gene expression vector in the *C. reinhardtii* FPKM data set, converting a set of ‘N’ FPKM values (x) in terms expression *vs.* time to new values (y) in terms of expression *vs.* frequency such that:

$$y_{k}=\sum_{n=0}^{N-1}x_{n}*e^{-i2\pi \frac{kn}{N}}$$ (1)

Where *x_n_* is the FKPM value at the nth time point and *y_k_* is the kth frequency component with period T/k, where T is the time period spanned by the expression vector. The set of frequency components represents the power spectra of the associated expression vector, that is, the contribution of each periodic cycle to the overall data. In calculating the power spectra of the expression data, we employed a nonwindowed application of Welch’s method (too few data points were present tolerate the loss of information involved in windowing) to average the power spectra over subsets of the expression vector with T = 24 hr. This was done to reduce bias in the calculation of the power spectra that might be induced by a particular subset of the expression data at the cost of reducing the overall resolution of the power spectra (although this loss of resolution was primarily at the extreme ends of the spectra and should not affect our results). Furthermore, the coefficients of each power spectra were normalized prior to averaging using the following equation:

$$y_{k}^{*}=\frac{y_{k}-y_{\min}}{y_{\max}-y_{\min}}$$ (2)

Where *y*_min_ is the smallest coefficient of the power spectra and *y*_max_ is the largest. As such, the normalized values,$y_{k}^{*}$, are on the interval [0,1], further reducing the affect that a single subset can have on the average power spectra. The “cyclic score” of each gene is defined as the normalized value of the 24 hr frequency component. This score is equated to a *P*-value by randomizing the order of values in each expression vector and scoring the vectors in this random population. For this study, we tested cyclic score thresholds equal to the fifth, second, and first percentiles of the score distribution of the randomized data (equal to 0.745, 0.808, and 0.841 respectively) and chose the second percentile as our cutoff for calling cycling genes (equivalent to a *P*-value of 0.02). In comparison, the fifth percentile of cyclic score for the set of predicted cycling genes in *C. reinhardtii* was 1. Additional information about how these thresholds were determined as well as a comparison to COPSPOT can be found in File S1.

### Clustering cycling genes according to phase

Cycling genes in *C. reinhardtii* were first divided by their phase of expression, that is, the ZT at which peak expression occurred in the FPKM data set. Within each phase cluster, genes were ordered using hierarchical clustering implemented in R for display purposes. Phase clusters were further broken down using two-rounds of k-means clustering, implemented using custom Python scripts. K-means clustering involves initially selecting “k” random centers in parameter space and assigning genes to clusters based on their distance to the nearest center. The mean of each cluster is then used to define new centers which in turn are used to redefine clusters; this process is repeated until the clusters converge or the amount change per iteration falls below a specified threshold. The final clusters used for pCRE identification contained 10−90 genes. Enrichment of Gene Ontology (GO) terms and pCREs in phase groups was done using the Fisher exact test, and the resulting *P*-values were corrected for multiple hypothesis testing using the method of Benjamini-Hochberg (Benjamini and Hochberg 1995).

### Conservation of cyclic expression and phase of expression among duplicate genes

Gene trees in *C. reinhardtii* were defined using the pipeline described in Zou *et al.* (2009) using a set of protein domains defined using PFAM (Punta *et al.* 2012). These domains were extracted from protein sequences and aligned using MAFFT (Katoh *et al.* 2002), and a phylogeny was inferred using RAxML (Stamatakis 2006) with parameters -f d -m PROTGAMMAJTT. Large domain families were divided by building neighbor joining trees with PHYLIP (Felsenstein 2005) and cutting a at distance to root ≥ 0.05 to create subclusters between 4 and 300 genes in size. Domains were mapped back to *C. reinhardtii* genes to infer gene trees. The gene trees, including the divided trees for large domain families, were reconciled with an existing species tree (Moreau *et al.* 2012) using NOTUNG (Chen *et al.* 2000). An archive of these gene trees in Nexus (.nex) format has been included as File S2. Branches containing *A. thaliana* and *C. reinhardtii* genes were extracted from the overall tree. The significance of the retention rate of cyclic expression and the phase of cyclic expression was determined by randomly pairing genes in the set of duplicates 100,000 times and comparing retention among actual duplicates to the random population.

### Modeling cycling state divergence of duplicate genes

The divergence of duplicate genes was modeled using a system of three difference equations with a common rate ‘d’ for the divergence of both cycling and noncycling duplicates and a common rate ‘s’ for the reversion of diverged duplicates back to an identical state. Duplicate gene pairs were binned according to *Ks* (width = 0.3), and we assume that the initial frequency of duplicates was the same within each bin (if the initial conditions were significantly different, we would expect to see deviation from the observed frequencies in the model predictions, which was not the case). We then solved for values of ‘d’ and ‘s’ using the observed change between consecutive bins, arriving at a solution with the same qualitative behavior as the observed data. A detailed description of the model can be found in File S1.

### Identification of pCREs and phase prediction

Identification of pCREs in the promoter regions of *C. reinhardtii* genes followed the pipeline described in Zou *et al.* (2011). Cycling genes were clustered according to phase and expression profile as previously described. For each cycling gene the promoter region, defined as the first 1-kb upstream of the transcription start site less any bases that overlap with another gene, was isolated. Six motif finders, AlignAce, MDscan MEME, Motif Sampler, Weeder, and YMF, were used to identify motifs enriched in the promoter region of each phase cluster compared with the promoters of all cycling genes. The resulting motifs were merged using UPGMA to reduce the number of motifs and remove redundant motifs. Merged motifs were mapped back to the *C. reinhardtii* genome using a threshold *P*-value of 1e-05.

The presence or absence of pCREs was used to predict the phase of expression of cycling genes using a support vector machine (SVM) implemented in Weka (Hall *et al.* 2009). Given a test-set of positive and negative examples defined using n-variables (in this case, presence or absence of pCREs), SVM seeks to define a linear classifier (*i.e.*, a hyperplane in variable space), that best divides positive and negative examples. This classifier is then used to assign subsequent data points to either the positive or negative set. A grid search of two parameters, the minimum distance between positive and negative groups (C) and the ratio of negative to positive examples in the training set (R), was used to optimize separation and pick the best classifier. The tested range of each parameter was as follows: C = (0.01, 0.1, 0.5, 1, 1.5, 2.0) and R = (0.25, 0.5, 1, 1.5, 2, 2.5, 3, 3.5, 4). Results were validated using 10-fold cross validation, which involved dividing positive and negative examples for each phase into training test sets using stratified random sampling. Each of the 10 test sets was classified by an independent SVM run and the average of the 10 runs was used to score the performance of the parameter set.

### Identifying groups of genes with common expression or common function

Cyclic genes with common expression were defined using k-means clustering as described previously. Cyclic genes with common function were defined as those that shared the same GO annotation. For the purpose of predicting cyclic expression, we used only those GO annotations overenriched in at least one phase of cyclic expression and where at least eight annotated genes were overenriched in the same phase.

## Results

### Cycling gene expression is extensive in the *C. reinhardtii* genome

To characterize cyclic expression in *C. reinhardtii*, the expression profiles of 17,114 annotated *C. reinhardtii* genes were defined from samples taken at 3-hr intervals over two 24-hr time courses (see the section *Materials and Methods*). A gene was defined as cyclically expressed if it exhibited statistically significant, nonrandom variation at a regular period as identified by either COSPOT or DFT (see the section *Materials and Methods*). The union of predictions for both methods covered 8072 cyclically expressed genes (47.2% of the *C. reinhardtii* genome), which we hereafter refer to as “cycling genes.” Both approaches generated cyclic expression models that correlated with the original expression data, with an average Pearson correlation coefficient of 0.987 for COSPOT and 0.880 for DFT. The correlation for COSPOT models is greater compared with that of DFT because COSPOT models are fit directly to the overall pattern of the data whereas the DFT models are based only on variations that occur at a period of 24 hr. Taken together, cyclic variation in gene expression represented the predominant form of nonlinear variation in RNA content at both the genome-wide and individual gene level.

**Figure 1:**
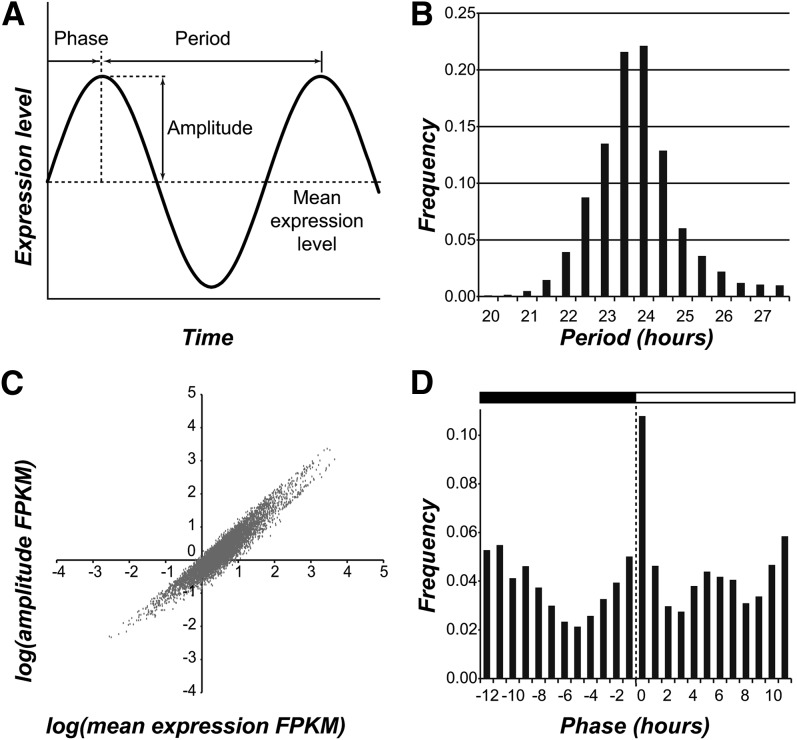
Period, amplitude, and phase of cyclic expression. (A) Three properties of cyclic variation: period, amplitude, and phase. (B) The distribution of period of cycling genes identified in *C. reinhardtii*. (C) The relationship between amplitude and mean expression level in FPKM (fragments per kilobase of transcript per million mapped reads). (D) The distribution of the phase of cycling genes.

Cyclic variation can be described using three parameters: period, amplitude, and phase (Figure 1A). Using the fitted models, we inferred the period, amplitude, and phase of all cycling genes in the *C. reinhardtii* genome. The distribution of period for our set of cycling genes was centered around 24 hr (+/− 1.10 hr, 95% confidence interval; Figure 1B and Figure S2A). The amplitude of cyclic expression was highly correlated with mean expression level (*r*^2^ > 0.7) and, on average, was only half the size of the mean, indicating that most cycling genes are expressed at some constitutive level even during the trough of the cycle (Figure 1C and Figure S2B). The phase distribution of cycling genes was bimodal with one peak at around ZT 0 (20.6% of cycling genes) and a second around ZT 12 (16.4% of cycling genes), corresponding to the night-to-day and the day-to-night transitions, respectively (Figure 1D and Figure S2C). Our finding concurs with the phase distribution reported for *A. thaliana* and other plant species under diel conditions (Michael *et al.* 2008; Filichkin *et al.* 2011) as well as a subset of circadian genes in *C. reinhardtii* (Kucho *et al.* 2005).

### Phases of cycling gene expression are associated with a succession of biological functions

**Figure 2:**
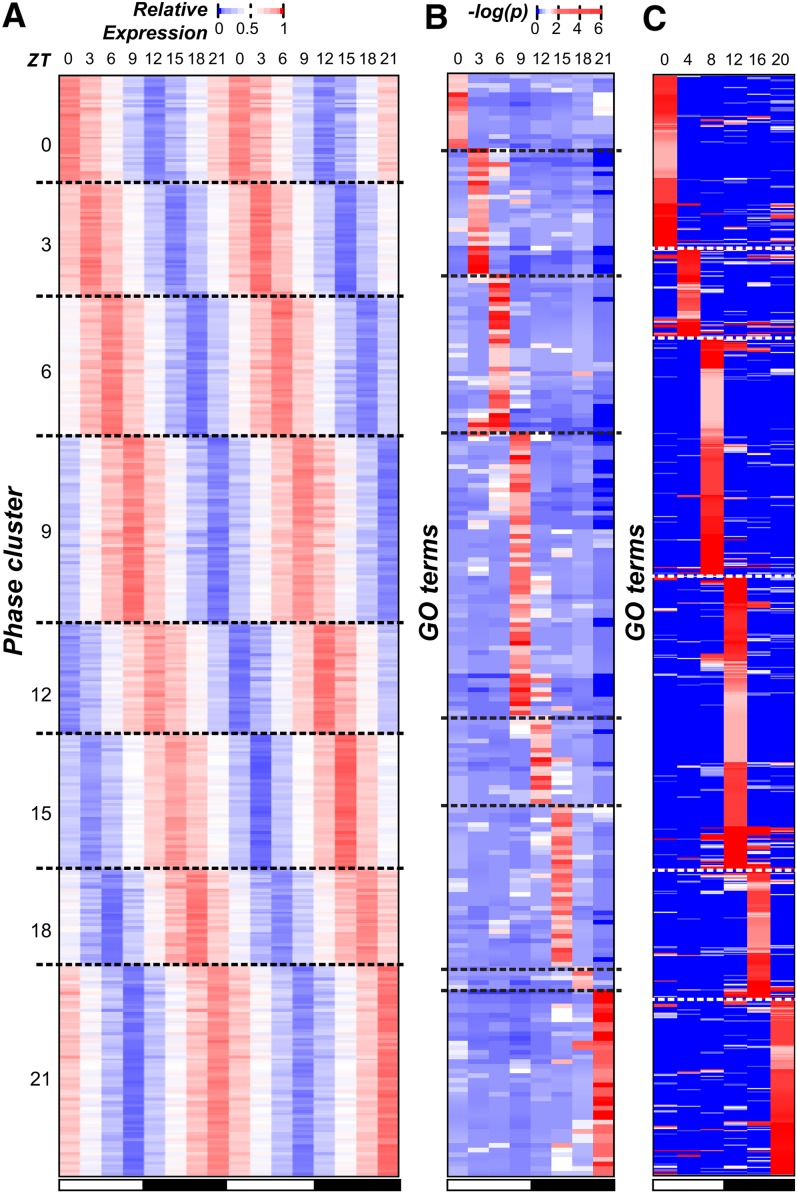
Phase of gene expression and cyclically expressed GO terms. (A) The normalized (relative) expression of each cycling gene in *C. reinhardtii* (each row) across the 48-hr period (columns). Genes were assigned to phase clusters based on the predicted time of peak expression. Genes in each phase cluster were ordered using hierarchical clustering. The white and black bars below indicate samples from the light and the dark periods, respectively. (B) The test statistics of GO term (rows) enrichment in each phase (columns) in *C. reinhardtii*. The −log(*p*-value) of the Fisher exact test is plotted. GO terms are ordered along the y-axis according to the most enriched phases and hierarchically clustered within each phase. (C) The test statistics of GO term enrichment in each phase in *A. thaliana*. Methods for assigning GO terms to phase, clustering, and the color legend are the same as in (B). GO, Gene Ontology.

Earlier studies have shown that multiple processes in *C. reinhardtii* have specific rhythms, including the expression of key photosystem components (Hwang and Herrin 1994; Jacobshagen and Johnson 1994) and the timing of gametogenesis (Jones 1970). Thus, we first asked which processes tend to be rhythmic by identifying GO terms with an overrepresented number of cycling genes. We found that cycling genes were enriched in 44 GO terms, including those related to the chloroplast, photosynthesis, and ribosomal subunits (Table S1). Among these terms, the most striking pattern was that 207 of 252 flagella related genes showed cyclic expression. In particular, 80% of cyclically expressed flagella genes (167 of 207) had peak expression at ZT 21, suggesting that biological functions can be phase specific. To further explore the association between phase and function, cycling genes were assigned to eight “phase clusters” (ZT 0, 3, 6, 9, 12, 15, 18, and 21; Figure 2A), and enrichment of GO categories within each cluster was determined.

We found that 237 GO terms had overrepresented numbers of genes in ≥1 phase cluster (Figure 2B). Enrichment values for each term in each phase group can be found in File S3. The greatest number of enriched terms was found in the ZT 21 cluster, just before the night-day transition, (40/237, 16.7%) and the ZT 9 cluster, just before the day-night transition (61/237, 25.7%). We also observed that overrepresented GO terms tended to be phase-specific: of all 237 terms, only 19 were enriched in >1 phase, and 12 of those were enriched only in two adjacent phases (Figure 2B and Figure S3A). In contrast, the majority of underrepresented categories (51%) spanned ≥4 phases (Figure S3B). Thus, genes involved in the same process not only tended to be enriched in a particular phase of expression but were also depleted in other phases. This phase-specificity of functional categories was consistent with previous studies of light-response, metabolism, cell division, and flagellum biogenesis in *C. reinhardtii* demonstrating cyclic behavior at a specific time of the day (Jones 1970; Cavalier-Smith 1974; Teramoto *et al.* 2002). For example, DNA replication and mitotic events in *C. reinhardtii* are restricted to the early hours of the dark period (Jones 1970): not only is the transition into darkness required for normal cell division (Voigt and Munzner 1987), but DNA replication and cell separation occur between 2 and 5 hr after the light-dark transition (Fang *et al.* 2006). This specific timing of DNA replication after the light to dark transition matches the phase of expression for cycling genes related to this process. Alternatively, the gradual increase in expression of replication associated genes toward a peak early in the dark period may track with increases in cell size, as it has been shown that the concentration of cell cycle regulatory proteins HA-MAT3, DP1, and E2F1 remain constant despite the increase in cell volume during G1 (Olson *et al.* 2010). We should note that many of the phase-specific functional categories uncovered here, such as amino acid biosynthesis, phosphorelay activity, and mRNA splicing were not previously known to show time-specific cycling behavior in *C. reinhardtii*. Although correlation alone is insufficient to prove causation, the coordination between cyclic expression and function is highly suggestive that timing of transcription can regulate the timing of higher order biological processes.

**Figure 3:**
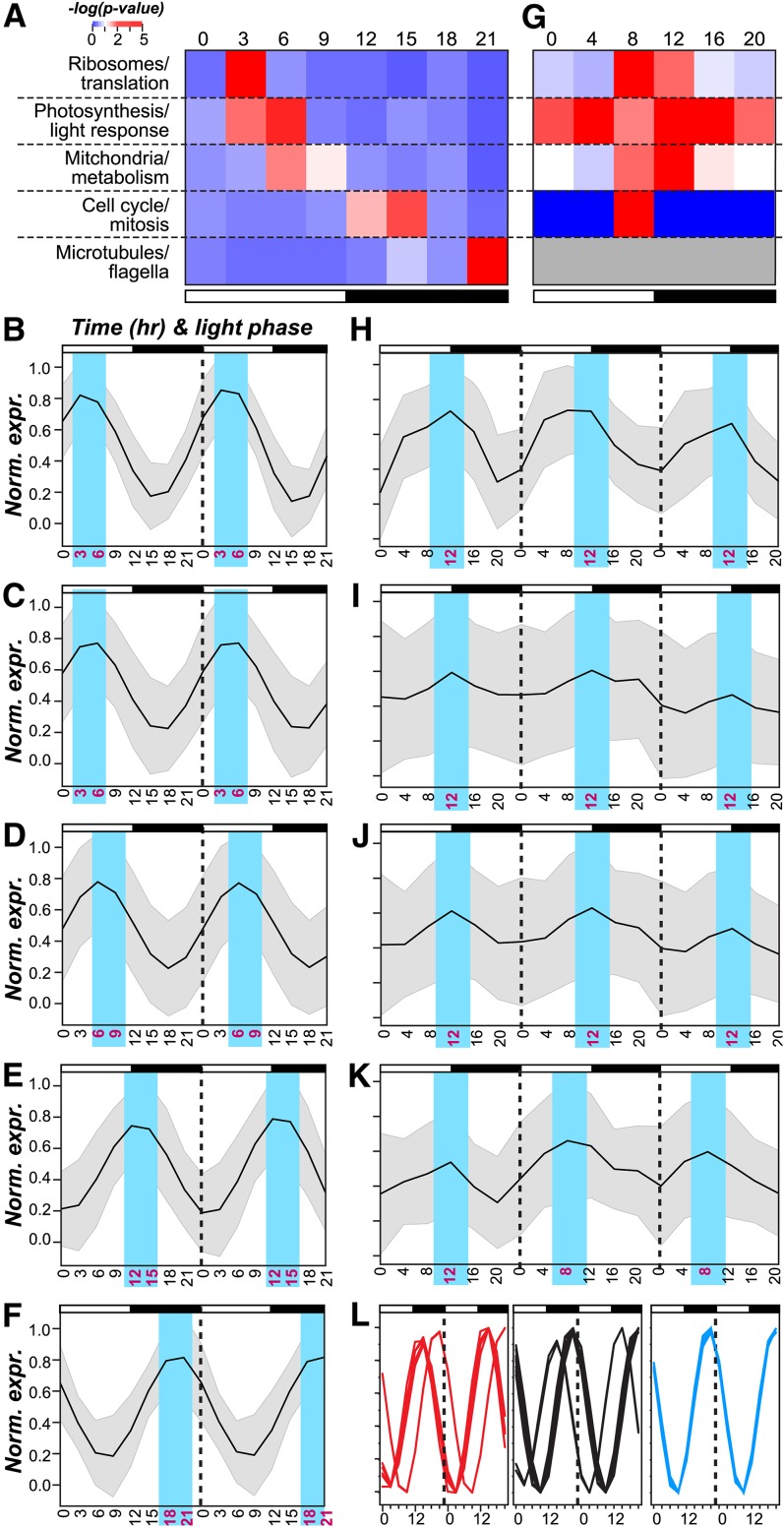
Phase specific expression of broad functional categories. (A) Enrichment test statistics in each functional group (row) and in each phase cluster (column) among *C. reinhardtii* cycling genes. The color indicates the averaged –log(*p*-value) of GO terms in a functional group Table S2 (B−F) Normalized expression profiles of genes in each functional group in *C. reinhardtii*. The black line indicates average expression values. The gray area represents +/−1 SD. (B) Ribosomes/translation; (C) photosynthesis/light-response; (D) mitochondria/metabolism; (E) cell-cycle/Mitosis; (F) microtubules/flagella; (G) enrichment test statistics for functional groups in *A. thaliana*. The functional group designation and color legends are the same as (A). Gray: not applicable. (H-K) Normalized expression profiles of genes in each functional group in *A. thaliana*. (H) Ribosomes/translation; (I) photosynthesis/light-response ;(J) mitochondria/Metabolism; (K) cell-cycle/mitosis; (L) expression profiles of genes in the microtubule cytoskeleton (red), flagellum assembly (blue), and cell projection organization (black) categories.

Based on the apparent association between phase and function in this as well as in previous studies, GO terms were classified into broad “functional groups”: (1) ribosome and translation, (2) photosynthesis and light response, (3) mitochondria and metabolism, (4) cell cycle and mitosis, and (5) microtubules and flagella (Figure 3 and Table S2). We found that group 1, 2, and 3 were overrepresented in the middle of the day (ZT 3 and 6), group 4 in the early and mid-night (ZT 12 and 15), and group 5 at the end of the dark-period (ZT 21) (Figure 3A). Consistent with the pattern of phase-specific enrichment of genes in different functional groups, the normalized expression profiles of cycling genes in each functional group clearly demonstrated phase specificity (Figure 3, B−F). The diel expression data also highlighted the possibility of distinguishing different components of a biological process. For example, group 5 genes are involved in forming microtubules and subsequently flagella. Within this group, genes associated with the microtubule cytoskeleton peaked earlier in the dark period whereas those associated with flagellum assembly peaked toward the end (Figure 3L), representing a clear delineation between spindle body formation and flagellar regeneration as described previously (Cavalier-Smith 1974). Taken together, our findings suggest that the timing of biological processes (translation, cell-replication, and regeneration of the flagellum) may be determined by transcriptional regulation.

### *C. reinhardtii* and *A. thaliana* orthologs have limited conservation in cycling gene expression patterns

To test whether the functional coordination and phase specificity of cyclic expression observed in *C. reinhardtii* can be found in related multicellular species, cycling genes were identified in *A. thaliana* using the same methods and cutoff values applied to *C. reinhardtii* on an existing diel expression data (Blasing *et al.* 2005). A total of 4945 genes in *A. thaliana* were identified as cycling (21.7% of the annotated genes), less than half of what was seen in *C. reinhardtii*. This difference is in part due to a lower sampling density of the *A. thaliana* data (once every 4 hr), though the overall time span covered was longer (3 d). It is also possible that the mixture of different cell types in *A. thaliana* samples could mask some rhythmic expression patterns. We also observed that 992 GO terms in *A. thaliana* were overrepresented in ≥1 phases compared with 237 in *C. reinhardtii*, which is likely a function of significantly better annotation (Figure 2C). Enrichment values for each term in each phase group can be found in File S4.

In contrast to the strict phase-specificity in *C. reinhardtii*, *A. thaliana* group 2 GO terms (photosynthesis and light response) were enriched among cycling genes in all six time points but were predominant at ZT 4. The other three groups (group 1, 3, and 4) were restricted to at most two adjacent phases (Figure 3G). Compared with *C. reinhardtii*, there is a greater variance in the phase of expression among the *A. thaliana* cycling genes within each group, potentially due to the fact that the *A. thaliana* expression data were derived from samples of mixed tissues and cell types. Nonetheless, the peak expression of photosynthetic, mitochondrial, and ribosomal genes occurred at a similar time, as was observed in *C. reinhardtii* (Figure 3, H−K). These results suggest that cyclic expression is conserved between a subset of functionally related genes, in both unicellular and multicellular plant systems.

Because of the concern that the phase-specificity differences between *C. reinhardtii* and *A. thaliana* might be due to annotation quality difference, we next examined the degree to which cycling gene expression was conserved between orthologous genes in these two species. Among 11,845 putative orthologs, 1464 (12.4%) showed cyclic expression in both species (referred to as “co-cycling” orthologs), which is significantly greater than the random expectation (χ^2^ test, *P* < 0.001). The conserved co-cycling genes encode components of the ribosome (particularly the small subunit), plasma, and thylakoid membrane components, or are involved in stress response (Fisher exact tests, *P <* 0.05). Nonetheless, we should emphasize that the difference between the observed and expected proportion of co-cycling orthologs was only 2.4%. Thus, most cycling genes in *C. reinhardtii* are not cyclic in *A. thaliana* and vice versa. In addition, although the amplitude of cyclic expression is significantly correlated among co-cycling orthologs (*r*^2^ = 0.30, *P <* 10^−100^), there are only weak relationships between their phases (*r*^2^ < 0.01, *P* < 0.006). The *A. thaliana* and *C. reinhardtii* lineages diverged 650−800 million years ago (Sanderson *et al.* 2004) and have extensive differences in life histories, distribution, complexity, and physiology. Thus, the conserved components of cyclic expression are likely core processes strongly selected to be maintained, including photosynthetic, mitochondrial, and ribosomal genes (Figure 3, H−K). However, most orthologs between green algae and flowering plants have divergent patterns of cyclic expression, and the extent of cyclic expression divergence highlights the fact that cycling gene expression can be plastic.

### Conservation of cyclic expression is more prevalent among older duplicate genes

**Figure 4:**
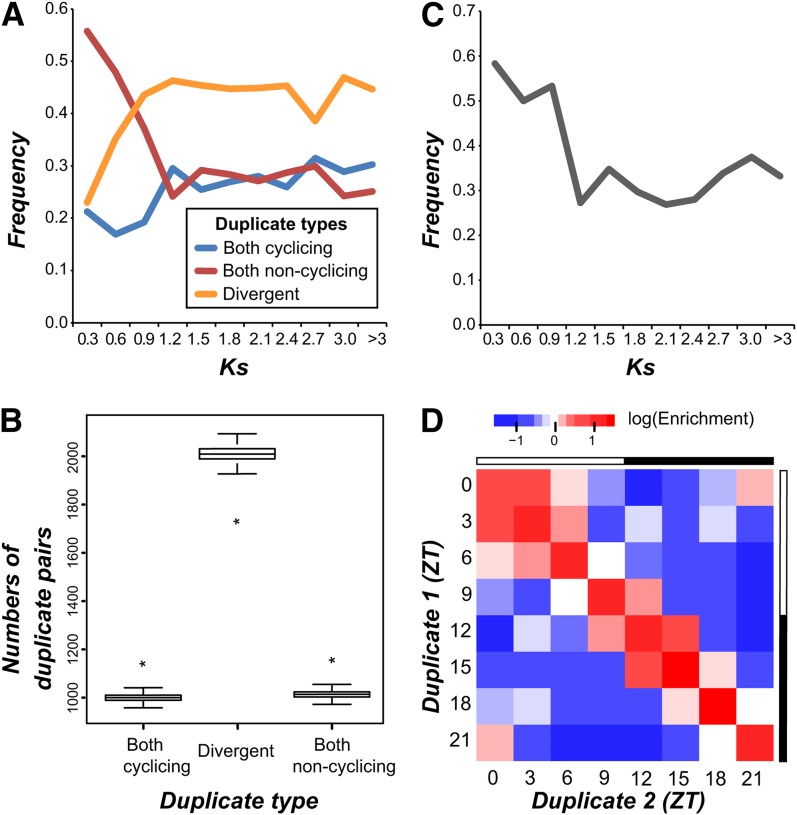
Conservation of cyclic expression and phase of cyclic expression. (A) The frequency at which duplicate pairs of genes in *C. reinhardtii* maintain cycling expression, maintain noncycling expression, or diverge as a function of the synonymous substitution rate (Ks). (B) Distribution of cycling retention, noncycling retention, and divergence between duplicate pairs in random simulations. The black bars cover the interquartile range of each distribution, and error bars represent the 95% confidence interval. Observed values are indicated by asterisks. (C) The frequency at which the phase is retained in pairs of cycling duplicates as a function of Ks. (D) Enrichment values for phase retention (diagonal values) and phase change (off diagonal values) between actual duplicates and duplicate pairs in random simulations.

To further assess how quickly cyclic expression divergence occurred, we asked how the pattern of cycling gene expression evolved between duplicated genes in *C. reinhardtii*. Gene trees were inferred based on similarity of known protein domains, and we retained only the closet pairs of paralogs (*i.e.*, those separated by only a single ancestral node) for subsequent study (see the section *Materials and Methods*). The frequency with which the pattern of gene expression (cycling or noncycling) was identical or divergent was compared with the timing of the inferred duplication event, estimated using the synonymous substitution rate (*Ks*) (Figure 4A). The overall frequency of diverged duplicates (one paralog cycling, the other noncycling) increased with *Ks*, approaching an asymptote of ~0.45 for *Ks* > 0.9. Although the frequency of noncycling duplicates decreased with *Ks*, the frequency of cycling duplicates was greater on average for *Ks* > 0.9 indicating a net gain of cycling expression as duplicates age. We hypothesized that this gain of cycling expression results from a bias in the rate at which duplicate genes diverge that favors the cycling state.

To test this hypothesis, we examined whether the observed changes in the frequency of retention can be explained without assuming different rates of divergence. Therefore, a null model of duplicate gene divergence was created using a system of difference equations (see the section *Materials and Methods*). We fit the transition probabilities using the difference in frequencies between *Ks* 0.6 and 0.9, and the predicted frequencies of identical and divergent duplicates closely matched our observed results at all time points (root mean squared error = 0.03), showing the same pattern of increases and decreases (Figure S4). Hence, we have no evidence of a differential rate in divergence between cycling and noncycling duplicates, however the predicted probability of transition from identical to divergent (0.42) is less than the probability of transition from divergent to identical (0.53), suggesting that there is a preference for maintaining duplicates in an identical state. This finding is consistent with our finding that the observed frequency of paralogs with identical states tends to be significantly greater than expected under random association (Z-test, *P* < 10^−17^; Figure 4B). In contrast, the frequency of paralogs with divergent state is significantly lower than expected (Figure 4B).

Next, we examined the frequency with which phase is identical among pairs of the cycling duplicates. Overall, the number of co-cycling paralogs for which the phase of cyclic expression was identical is more than twice the number randomly expected (Z-test, *P* < 10^−85^) with 33.7% of co-cycling duplicates sharing the same phase. The identical phase state was more common among cycling duplicates with lower *Ks*, and there was a sharp decrease in the frequency of duplicates with identical phases going from a *Ks* of 0.9 to 1.2 (Figure 4C). Next we explored whether there was a bias in the magnitude of phase change between co-cycling duplicates (Figure 4D). We found that small phase divergences of +/− 3 hr (covering 28.3% of all duplicates) tended to be enriched relative to random expectation, in particular at ZT0/ZT3 and ZT12/ZT15, although the identical phase state is still the most highly enriched scenario. Additionally, there was an inverse, linear relationship between the magnitude of the difference in phase between cycling duplicates and the enrichment of phase-shift events relative to random expectation (all cycling duplicates, *r*^2^ = 0.91; duplicates with *Ks* > 0.9, *r*^2^ = 0.93), indicating that large differences in phase between duplicates occur less frequently than expected by random chance. Furthermore, we found 33 GO terms enriched (adjusted *P*-value < 0.05) among cycling duplicates with the same phase, the majority of which (88%) were previously found to be enriched in a specific phase of cyclic expression.

### Cycling genes are enriched for specific pCREs

**Figure 5:**
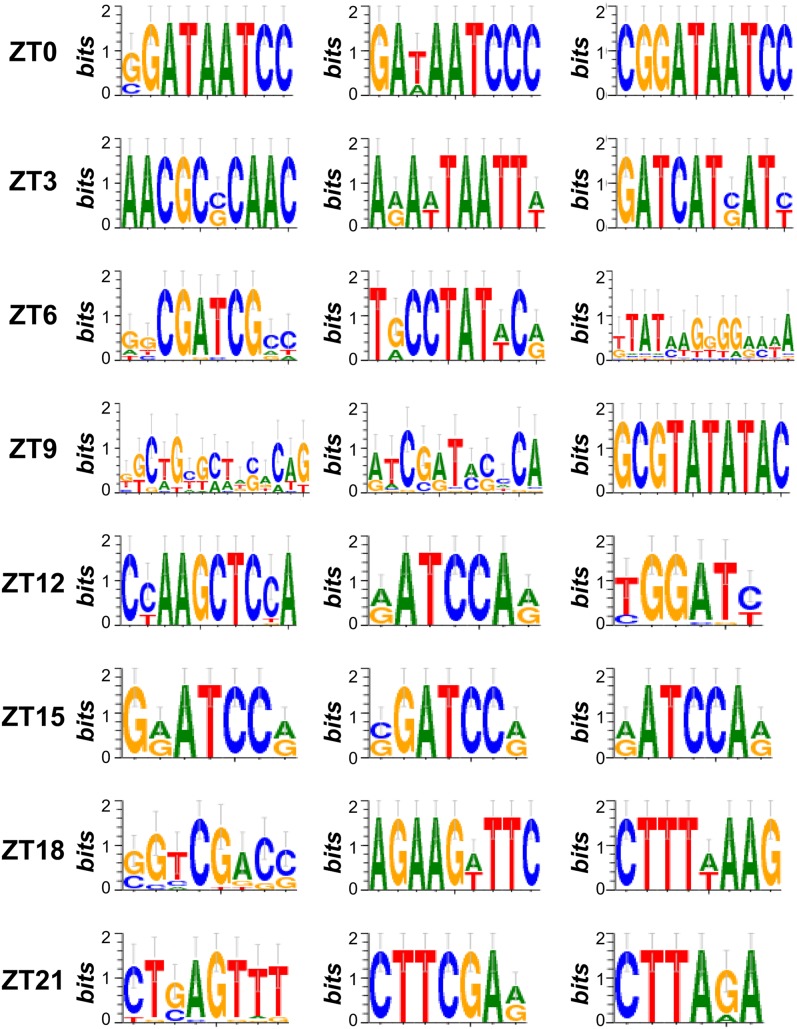
Top three pCREs enriched in each phase cluster of cyclic genes. Sequence logos representing the top three putative *cis*-regulatory elements (pCREs) enriched in each phase cluster of cycling genes in *C. reinhardtii*.

**Figure 6:**
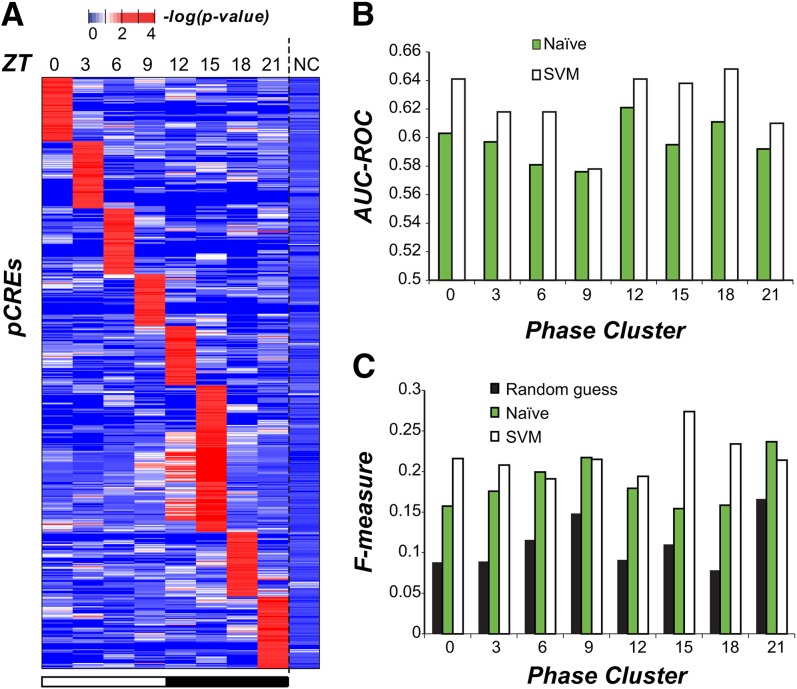
Enrichment and performance of phase-specific pCREs. (A) The enrichment test statistics of 687 pCREs (rows) in genes of each phase cluster and noncyclic genes (columns). (B) The area under the curve of the receiver operating characteristic (AUC-ROC) for phase expression prediction with naïve (green) and support vector machine (SVM, white) classifiers. (C) The F-measures for phase expression prediction based on random guess (black), naïve (green), and SVM (white) classifiers.

The coordinated expression of functionally related genes suggests the existence of one or more regulatory mechanisms that drive phase specific expression. Although mRNA levels may be affected at multiple levels of regulation, we chose to focus on transcriptional regulation driven by *cis*-regulatory sequences as circadian rhythm related *cis*-elements have previously been identified in plant and animal models (Michael and McClung 2002; Ueda *et al.* 2005; Michael *et al.* 2008). Using a motif finding pipeline (Zou *et al.* 2011), we found 687 pCREs in the 1-kb regions upstream of the transcriptional start sites of cycling genes for each of the eight *C. reinhardtii* phase clusters (Fisher exact test, adjusted *P* < 0.05). The top enriched motifs for each phase can be found in Figure 5, and the entire list of enriched motifs can be found in File S5. Each phase had 60-84 associated pCREs, except for ZT 15 with 169; however, more than 20% of pCREs (141/687) were enriched in >1 phase and 43.8% of ZT 15 pCREs (74/169) were enriched in ≥1 other phases (mostly ZT 12; Figure 6A). Therefore, each pCRE was assigned to the phase cluster in which it was most significantly enriched.

To further assess whether the pCREs are meaningful, they were used to establish classifiers to predict cyclic expression in different phases. First, the pCREs assigned to each phase were used to predict which genes are cyclic in a naïve manner. That is, for pCREs enriched in a particular phase, we simply predicted that all genes with ≥1 pCREs mapped to their promoters would cycle at that phase. The performance of these predictions was assessed using the area under the receiver operating curve (AUC-ROC), a metric that quantifies the ability of a method to predict positive examples which, in our case, is phase specific expression. Perfect predictors have an AUC-ROC of 1, whereas random guessing has a value of 0.5; our naïve classification of phase had AUC-ROCs that ranged from 0.58 (ZT 9) to 0.62 (ZT 12) indicating that this simple classification procedure performed marginally better than randomly assigning phase (Figure 6B). The same conclusion can be reached based on the F-measure, another prediction performance metric (Figure 6C). Next, to further improve the prediction of the phase of cyclic expression, we used the SVM algorithm to classify cycling genes according to the presence or absence of all pCREs (see the section *Materials and Methods*). The SVM classifier shows improved performance compared to naïve classification (Figure 6, B and C and Table S3) but AUC-ROC values are still relatively low, ranging from 0.58 (ZT 9) to 0.65 (ZT18) (Figure S5). We also identified two pCRE association rules enriched in specific phases of cyclic expression using CBA (Liu *et al.* 1998); however, adding these rules to the SVM prediction models did not significantly improve the overall predictive power of our pCREs as the AUC-ROC increased by at most 0.01.

Given that the *C. reinhardtii* pCREs are computationally derived, we next asked how well a known, experimentally verified, phase-specific CRE may predict cyclic expression. For this purpose we examined the Evening Element that is necessary and sufficient to drive circadian expression in *A. thaliana* (Michael and McClung 2002; Harmer and Kay 2005; Michael *et al.* 2008). Using motifs related to the Evening Element identified in Michael *et al.* (2008), we generated a cycling gene classifier to predict the phase of the 4945 *A. thaliana* cycling genes introduced in the earlier phase-specificity comparison section. The optimal AUC-ROC of the Evening Element classifier was 0.57 and 0.56 at ZT 0 and 12 hr, respectively (compared with 0.58−0.65 in *C. reinhardtii* pCRE predictions). Therefore, although the Evening Element is known to function as a circadian regulator, similar to *C. reinhardtii* pCRES, it has only limited predictive power on a genome wide scale. To obtain accurate predictions the presence or absence of pCREs needs to be supplemented with additional information regarding the regulation of cycling expression.

### Phase of cyclic expression can be predicted for groups of genes with common expression patterns or common function

**Figure 7:**
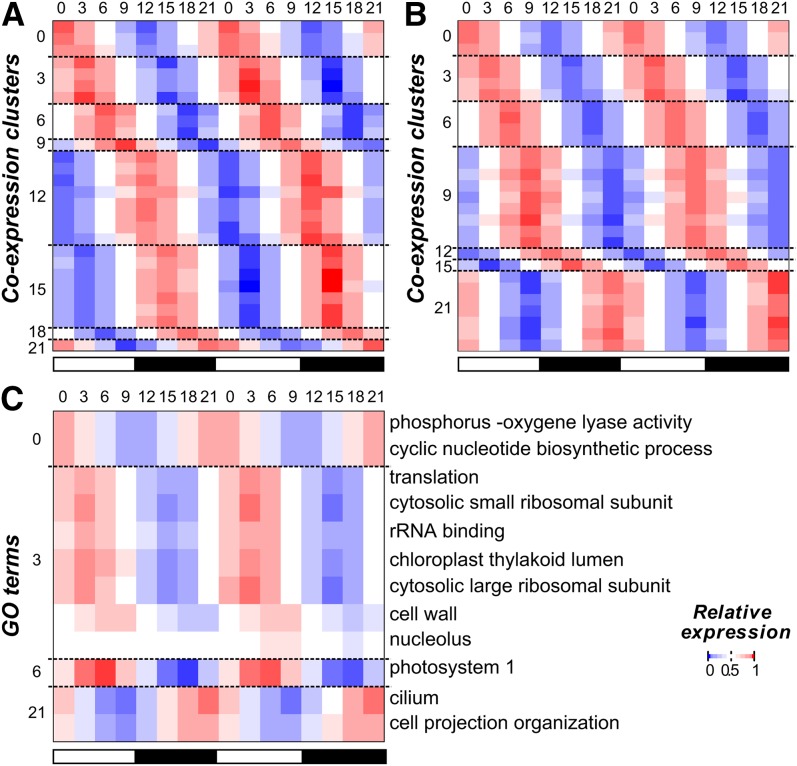
Expression of best predicted coexpression cluster and GO terms. (A) Averaged, normalized expression profile of genes in the top 28 coexpression clusters whose phase of expression can be predicted with AUC-ROC > 0.7. (B) Averaged, normalized expression profiles of genes in the bottom 28 coexpression clusters whose phase of expression can be predicted with AUC-ROC < 0.56. (C) Averaged, normalized expression profiles of genes in the 12 GO terms whose phase of expression can be predicted with AUC-ROC > 0.7. Both cycling and noncycling genes annotated in each GO term are included. GO, Gene Ontology.

The weak predictive power of pCREs likely results from an underlying complexity in the regulation of the phase of cyclic expression, either in the form of additional control mechanisms or the existence of more discrete regulatory groups. Timing of cyclic expression may be modified by interactions among regulatory motifs or posttranscriptional mechanisms. It is also possible that our phase clusters might consist of multiple regulatory subgroups. To address the latter possibility, we further classified genes in each phase group into subclusters containing genes with highly similar expression profiles (phase-expression clusters). Using SVM, 28 of 190 phase-expression clusters covering 584 genes (7.23% of cycling genes) could be classified with an AUC-ROC > 0.7 (these clusters are described in File S6), which is better than any individual phase alone. The best predicted phase-expression clusters do not necessarily have stronger cyclic signals (Figure 7A) compared with the worst predicted (Figure 7B). Additionally, we eliminated size (*r*^2^ = 0.15) and the correlation of expression profiles within each phase-expression cluster (*r*^2^ < 0.01) as possible variables explaining the observed variance in AUC-ROC (Figure S6). These results suggest that phase-specific regulation does occur at the *cis*-regulatory level for particular groups of cycling genes and that presence or absence of pCREs alone is sufficient to accurately predict the pattern of phase specific expression for these clusters. Those pCREs which were informative (*i.e.*, had the non-zero weights) when predicting the 28 best phase-expression clusters are listed in File S7.

In addition to using highly similar expression patterns as a way of subdividing phase clusters, we looked for evidence of phase specific regulation among groups of genes in the same phase cluster that had related annotated function (phase-function clusters). Among 71 phase-function clusters, genes belonging to 12 of these clusters could be classified with an AUC-ROC > 0.7. These clusters covered 12.2% (175/1434) of genes present in all phase-function clusters, a greater percentage than the phase-expression clusters, although they constitute a smaller portion of all cycling genes due to limited GO annotation in *C. reinhardtii* (these cluster are described in File S8). Genes in most of these functional groups displayed a clear cyclic signal (Figure 7C), except for the groups related to the nucleolus and cell wall, which were predominantly non-cyclic genes but had a statistically significant subset of phase-specific genes. Among the best classified subclusters contained genes relating to the large cytosolic ribosomal subunits (AUC-ROC = 0.73), cilium (0.72), small cytosolic ribosomal subunit (0.72), translation (0.71), and the chloroplast (0.68). This supports our earlier observation that the cyclic patterning of large scale processes such as photosynthesis, translation, and motility may be regulated at the transcriptional level. The pCREs which had non-zero weights when predicting the 12 best phase-function clusters are listed in File S9.

## Discussion

We have determined that cyclic expression is prevalent in the *C. reinhardtii* genome, and nearly half of all annotated genes cycle under diel conditions. There is a strong link between rhythmic patterns at the molecular and physiological levels. Diel cycling expression is influenced both by environmental factors, such as the availability of light, and endogenous factors, including metabolism and the circadian clock (Farre 2012; Kinmonth-Schultz *et al.* 2013; Song *et al.* 2013; Fonken and Nelson 2014). Although the importance of photoperiod can be inferred for light-dependent (*i.e.*, photo-synthesis) and light-sensitive (*i.e.*, DNA replication) processes, for most cycling related functions it remains an open question as to what extent each factor influences cycling expression. This is particularly true of functions which were not previously known to exhibit cycling expression in green algae, for example, the regulation of RNA processing and amino-acid synthesis.

In addition to the relationship between cyclic expression and gene function, we found that cyclic expression was significantly conserved between paralogous genes. The proportion of divergent duplicates reaches an asymptote at Ks > 0.9, which is similar to what was previously observed for stress responsive duplicate genes (Zou *et al.* 2009). However, although there appears to be a clear preference for the partitioning of ancestral expression states in stress responsive genes (Zou *et al.* 2009; Dong and Adams 2011), we found that duplicates genes tend to share the same expression state with respect to cycling and that cycling duplicates preferentially retain the same or similar phase of expression. We hypothesize this pattern of cyclicity/phase conservation among duplicates points to a fundamentally distinct regulatory logic from that of stress response. In stress response, a duplicate that has lost response to one condition may still be responsive to other conditions and thus retained. However, either loss or gain of cyclicity in a duplicate gene would mean it is no longer temporally in sync with other genes in the processes which it was originally involved in. For example, if a replication initiation factor duplicate was not in sync with the expression of other components of the replication machinery, the duplicated factor would not be functional and eventually eliminated from the genome. This argument may also apply to the conservation of phase among duplicate cycling genes. Indeed, we found that most GO terms enriched among co-cycling duplicates with the same phase were highly phase specific, including those associated with DNA replication and flagellar components.

Based on previous studies of stress response genes (Zou *et al.* 2009), we expected that the conservation of cycling expression state, particularly the phase of expression, would be correlated with the presence of shared CREs. However, contrary to this expectation, the set of putative CREs enriched in cycling genes does not accurately distinguish phase expression. Although our results suggest that *cis*-regulation plays a significant role in controlling cyclic expression in *C. reinhardtii*, the presence or absence of promoter elements alone was insufficient to fully explain the observed patterns of cyclic variation across the entire *C. reinhardtii* genome. This finding suggests that additional regulatory components are involved in controlling cyclic expression. In other organisms, the combinatorial interactions among regulatory factors play an important role in controlling the phase of cyclic gene expression (Harmer and Kay 2005; Ueda *et al.* 2005), but in *C. reinhardtii* there is evidence that response to changing light levels is mediated by multiple copies of the same or similar promoter elements (von Gromoff *et al.* 2006). Although we did not see significant improvement when rules considering combinatorial relationships between pCREs were included in our model, this may be due to the fact that we were able to explore only a subset of all possible combinatorial interactions in our pCRE set. Additionally, posttranscriptional regulation has been implicated in regulating circadian processes in *Neurospora crassa*, *A. thaliana*, and *D. melanogaster* (Kojima *et al.* 2011). In *C. reinhardtii*, the over- or underexpression of the RNA-binding protein CHLAMY1 is known to result in the disruption or loss of circadian rhythms (Iliev *et al.* 2006). Further studies incorporating posttranscriptional regulatory features will be necessary to improve the prediction of phase-specific cyclic expression.

The inability of pCREs to classify phase specific cycling expression on a genome-wide scale does not contradict previous observations that certain *cis*-elements are necessary for cycling expression (Michael and McClung 2002; Harmer and Kay 2005; Michael *et al.* 2008). Rather it suggests that *cis*-elements alone are insufficient to explain the variation in cycling expression on a genome wide scale and that additional regulatory components remain to be discovered. Posttranscriptional regulatory mechanisms and chromatin state are two promising avenues of investigation which, in conjunction with the *cis* elements we have identified, could be used to better predict the state of cycling expression. Although there remains substantial room for further improvement, our findings contribute to a better understanding of both the function and evolutionary origins of cyclic expression in a green alga, laying the foundation for further molecular dissection of the relationships between the rhythmic gene expression and physiological functions for potential biotechnological applications.

## Supplementary Material

Supporting Information

## References

[bib1] Arimura G. Kopke S. Kunert M. Volpe V. David A., 2008 Effects of feeding *Spodoptera littoralis* on lima bean leaves: IV. Diurnal and nocturnal damage differentially initiate plant volatile emission. Plant Physiol. 146: 965–973. 18165324 10.1104/pp.107.111088 PMC2259069

[bib2] Baldwin I. T. Meldau S., 2013 Just in time: circadian defense patterns and the optimal defense hypothesis. Plant Signal. Behav. 8: e24410. 23603968 10.4161/psb.24410 PMC3909060

[bib3] Benjamini Y. Hochberg Y., 1995 Controlling the false discovery rate—a practical and powerful approach to multiple testing. J. R. Stat. Soc., B 57: 289–300.

[bib4] Bisova K. Krylov D. M. Umen J. G., 2005 Genome-wide annotation and expression profiling of cell cycle regulatory genes in *Chlamydomonas reinhardtii*. Plant Physiol. 137: 475–491. 15710686 10.1104/pp.104.054155 PMC1065349

[bib5] Blasing O. E. Gibon Y. Gunther M. Hohne M. Morcuende R., 2005 Sugars and circadian regulation make major contributions to the global regulation of diurnal gene expression in Arabidopsis. Plant Cell 17: 3257–3281. 16299223 10.1105/tpc.105.035261 PMC1315368

[bib6] Bruce V. G., 1970 The biological clock in *Chlamydomonas reinhardtii*. J. Protozool. 17: 328–334.

[bib7] Bullard J. H. Purdom E. Hansen K. D. Dudoit S., 2010 Evaluation of statistical methods for normalization and differential expression in mRNA-Seq experiments. BMC Bioinformatics 11: 94. 20167110 10.1186/1471-2105-11-94 PMC2838869

[bib8] Byrne T. E. Wells M. R. Johnson C. H., 1992 Circadian rhythms of chemotaxis to ammonium and of methylammonium uptake in chlamydomonas. Plant Physiol. 98: 879–886. 16668759 10.1104/pp.98.3.879 PMC1080282

[bib9] Cavalier-Smith T., 1974 Basal body and flagellar development during the vegetative cell cycle and the sexual cycle of *Chlamydomonas reinhardii*. J. Cell Sci. 16: 529–556. 4615103 10.1242/jcs.16.3.529

[bib10] Chen K. Durand D. Farach-Colton M., 2000 NOTUNG: a program for dating gene duplications and optimizing gene family trees. J. Comput. Biol. 7: 429–447. 11108472 10.1089/106652700750050871

[bib11] Corellou F. Schwartz C. Motta J. P. Djouani-Tahri el B. Sanchez F., 2009 Clocks in the green lineage: comparative functional analysis of the circadian architecture of the picoeukaryote ostreococcus. Plant Cell 21: 3436–3449. 19948792 10.1105/tpc.109.068825 PMC2798331

[bib12] Davies J. P. Grossman A. R., 1994 Sequences controlling transcription of the *Chlamydomonas reinhardtii* beta 2-tubulin gene after deflagellation and during the cell cycle. Mol. Cell. Biol. 14: 5165–5174. 8035797 10.1128/mcb.14.8.5165-5174.1994 PMC359035

[bib13] Doherty C. J. Kay S. A., 2010 Circadian control of global gene expression patterns. Annu. Rev. Genet. 44: 419–444. 20809800 10.1146/annurev-genet-102209-163432 PMC4251774

[bib14] Dong S. Adams K. L., 2011 Differential contributions to the transcriptome of duplicated genes in response to abiotic stresses in natural and synthetic polyploids. New Phytol. 190: 1045–1057. 21361962 10.1111/j.1469-8137.2011.03650.x

[bib15] Fang S. C. de los Reyes C. Umen J. G., 2006 Cell size checkpoint control by the retinoblastoma tumor suppressor pathway. PLoS Genet. 2: e167. 17040130 10.1371/journal.pgen.0020167 PMC1599770

[bib16] Farre E. M., 2012 The regulation of plant growth by the circadian clock. Plant Biol (Stuttg) 14: 401–410. 22284304 10.1111/j.1438-8677.2011.00548.x

[bib17] Felsenstein, J., 2005 PHYLIP (Phylogeny Inference Package) version 3.6. Distributed by the author.

[bib18] Filichkin S. A. Breton G. Priest H. D. Dharmawardhana P. Jaiswal P., 2011 Global profiling of rice and poplar transcriptomes highlights key conserved circadian-controlled pathways and *cis*-regulatory modules. PLoS ONE 6: e16907. 21694767 10.1371/journal.pone.0016907 PMC3111414

[bib19] Fonken L. K. Nelson R. J., 2014 The effects of light at night on circadian clocks and metabolism. Endocr. Rev. 35: 648–670. 24673196 10.1210/er.2013-1051

[bib20] Frund J. Dormann C. F. Tscharntke T., 2011 Linne’s floral clock is slow without pollinators–flower closure and plant−pollinator interaction webs. Ecol. Lett. 14: 896–904. 21752170 10.1111/j.1461-0248.2011.01654.x

[bib21] Goodspeed D. Chehab E. W. Min-Venditti A. Braam J. Covington M. F., 2012 Arabidopsis synchronizes jasmonate-mediated defense with insect circadian behavior. Proc. Natl. Acad. Sci. USA 109: 4674–4677. 22331878 10.1073/pnas.1116368109 PMC3311395

[bib22] Grossman A. R. Croft M. Gladyshev V. N. Merchant S. S. Posewitz M. C., 2007 Novel metabolism in *Chlamydomonas* through the lens of genomics. Curr. Opin. Plant Biol. 10: 190–198. 17291820 10.1016/j.pbi.2007.01.012

[bib23] Hall M. Frank E. Holmes G. Pfahringer B. Reutemann P., 2009 The WEKA data mining software: an update. SIGKDD Explor. Newsl. 11: 10–18.

[bib24] Harmer S. L. Kay S. A., 2005 Positive and negative factors confer phase-specific circadian regulation of transcription in Arabidopsis. Plant Cell 17: 1926–1940. 15923346 10.1105/tpc.105.033035 PMC1167542

[bib25] Harris E. H., 2001 *Chlamydomonas* as a model organism. Annu. Rev. Plant Physiol. Plant Mol. Biol. 52: 363–406. 11337403 10.1146/annurev.arplant.52.1.363

[bib26] Hu Q. Sommerfeld M. Jarvis E. Ghirardi M. Posewitz M., 2008 Microalgal triacylglycerols as feedstocks for biofuel production: perspectives and advances. Plant J. 54: 621–639. 18476868 10.1111/j.1365-313X.2008.03492.x

[bib27] Hwang S. Herrin D. L., 1994 Control of lhc gene transcription by the circadian clock in *Chlamydomonas reinhardtii*. Plant Mol. Biol. 26: 557–569. 7948912 10.1007/BF00013743

[bib28] Iliev D. Voytsekh O. Schmidt E. M. Fiedler M. Nykytenko A., 2006 A heteromeric RNA-binding protein is involved in maintaining acrophase and period of the circadian clock. Plant Physiol. 142: 797–806. 16920878 10.1104/pp.106.085944 PMC1586056

[bib29] Jacobshagen S. Johnson C. H., 1994 Circadian rhythms of gene expression in *Chlamydomonas reinhardtii*: circadian cycling of mRNA abundances of cab II, and possibly of beta-tubulin and cytochrome c. Eur. J. Cell Biol. 64: 142–152. 7957302

[bib30] Jones R. F., 1970 Physiological and biochemical aspects of growth and gametogenesis in *Chlamydomonas-Reinhardtii*. Ann. N. Y. Acad. Sci. 175: 648–659.

[bib31] Katoh K. Misawa K. Kuma K. Miyata T., 2002 MAFFT: a novel method for rapid multiple sequence alignment based on fast Fourier transform. Nucleic Acids Res. 30: 3059–3066. 12136088 10.1093/nar/gkf436 PMC135756

[bib32] Kinmonth-Schultz H. A. Golembeski G. S. Imaizumi T., 2013 Circadian clock-regulated physiological outputs: dynamic responses in nature. Semin. Cell Dev. Biol. 24: 407–413. 23435352 10.1016/j.semcdb.2013.02.006 PMC3742325

[bib33] Kojima S. Shingle D. L. Green C. B., 2011 Post-transcriptional control of circadian rhythms. J. Cell Sci. 124: 311–320. 21242310 10.1242/jcs.065771 PMC3021995

[bib34] Kucho K. Okamoto K. Tabata S. Fukuzawa H. Ishiura M., 2005 Identification of novel clock-controlled genes by cDNA macroarray analysis in *Chlamydomonas reinhardtii*. Plant Mol. Biol. 57: 889–906. 15952072 10.1007/s11103-005-3248-1

[bib35] Lehmann M. Gustav D. Galizia C. G., 2011 The early bee catches the flower - circadian rhythmicity influences learning performance in honey bees, *Apis mellifera*. Behav. Ecol. Sociobiol. 65: 205–215. 21350590 10.1007/s00265-010-1026-9 PMC3022154

[bib36] Liu B. Hsu W. Ma Y., 1998 Integrating classification and association rule mining, pp. 80−86 in Proceedings of the Fourth International Conference on Knowledge Discovery and Data Mining Conference, edited by Agrawal R. Stolorz P. AAAI Press, Menlo Park, CA.

[bib37] Matsuo T. Ishiura M., 2010 New insights into the circadian clock in *Chlamydomonas*. Int Rev Cell Mol Biol 280: 281–314. 20797685 10.1016/S1937-6448(10)80006-1

[bib38] McNabb S. L. Truman J. W., 2008 Light and peptidergic eclosion hormone neurons stimulate a rapid eclosion response that masks circadian emergence in *Drosophila*. J. Exp. Biol. 211: 2263–2274. 18587121 10.1242/jeb.015818 PMC2760273

[bib39] Michael T. P. McClung C. R., 2002 Phase-specific circadian clock regulatory elements in *Arabidopsis*. Plant Physiol. 130: 627–638. 12376630 10.1104/pp.004929 PMC166592

[bib40] Michael T. P. Mockler T. C. Breton G. McEntee C. Byer A., 2008 Network discovery pipeline elucidates conserved time-of-day-specific *cis*-regulatory modules. PLoS Genet. 4: e14. 18248097 10.1371/journal.pgen.0040014 PMC2222925

[bib41] Mittag M. Kiaulehn S. Johnson C. H., 2005 The circadian clock in *Chlamydomonas reinhardtii*. What is it for? What is it similar to? Plant Physiol. 137: 399–409. 15710681 10.1104/pp.104.052415 PMC1065344

[bib42] Monnier A. Liverani S. Bouvet R. Jesson B. Smith J. Q., 2010 Orchestrated transcription of biological processes in the marine picoeukaryote Ostreococcus exposed to light/dark cycles. BMC Genomics 11: 192. 20307298 10.1186/1471-2164-11-192 PMC2850359

[bib43] Moreau H. Verhelst B. Couloux A. Derelle E. Rombauts S., 2012 Gene functionalities and genome structure in Bathycoccus prasinos reflect cellular specializations at the base of the green lineage. Genome Biol. 13: R74. 22925495 10.1186/gb-2012-13-8-r74 PMC3491373

[bib44] Olson B. J. Oberholzer M. Li Y. Zones J. M. Kohli H. S., 2010 Regulation of the Chlamydomonas cell cycle by a stable, chromatin-associated retinoblastoma tumor suppressor complex. Plant Cell 22: 3331–3347. 20978220 10.1105/tpc.110.076067 PMC2990127

[bib45] Pajuelo E. Pajuelo P. Clemente M. T. Marquez A. J., 1995 Regulation of the expression of ferredoxin-nitrite reductase in synchronous cultures of *Chlamydomonas reinhardtii*. Biochim. Biophys. Acta 1249: 72–78. 7766686 10.1016/0167-4838(95)00066-4

[bib46] Panda S. Antoch M. P. Miller B. H. Su A. I. Schook A. B., 2002 Coordinated transcription of key pathways in the mouse by the circadian clock. Cell 109: 307–320. 12015981 10.1016/s0092-8674(02)00722-5

[bib47] Punta M. Coggill P. C. Eberhardt R. Y. Mistry J. Tate J., 2012 The Pfam protein families database. Nucleic Acids Res. 40: D290–D301. 22127870 10.1093/nar/gkr1065 PMC3245129

[bib48] Ral J. P. Colleoni C. Wattebled F. Dauvillee D. Nempont C., 2006 Circadian clock regulation of starch metabolism establishes GBSSI as a major contributor to amylopectin synthesis in *Chlamydomonas reinhardtii*. Plant Physiol. 142: 305–317. 16844835 10.1104/pp.106.081885 PMC1557617

[bib49] Ratcliff W. C. Herron M. D. Howell K. Pentz J. T. Rosenzweig F., 2013 Experimental evolution of an alternating uni- and multicellular life cycle in *Chlamydomonas reinhardtii*. Nat. Commun. 4: 2742. 24193369 10.1038/ncomms3742 PMC3831279

[bib50] Rodriguez J. Tang C. H. A. Khodor Y. L. Vodala S. Menet J. S., 2013 Nascent-Seq analysis of *Drosophila* cycling gene expression. Proc. Natl. Acad. Sci. USA 110: E275–E284. 23297234 10.1073/pnas.1219969110 PMC3557077

[bib51] Romero J. M. Valverde F., 2009 Evolutionarily conserved photoperiod mechanisms in plants: when did plant photoperiodic signaling appear? Plant Signal. Behav. 4: 642–644. 19820341 10.4161/psb.4.7.8975 PMC2710563

[bib52] Rosa B. A. Jiao Y. H. Oh S. Montgomery B. L. Qin W. S., 2012 Frequency-based time-series gene expression recomposition using PRIISM. BMC Syst. Biol. 6: 69. 22703599 10.1186/1752-0509-6-69 PMC3464900

[bib53] Sanderson M. J. Thorne J. L. Wikstrom N. Bremer K., 2004 Molecular evidence on plant divergence times. Am. J. Bot. 91: 1656–1665. 21652315 10.3732/ajb.91.10.1656

[bib54] Shi T. Ilikchyan I. Rabouille S. Zehr J. P., 2010 Genome-wide analysis of diel gene expression in the unicellular N(2)-fixing cyanobacterium *Crocosphaera watsonii* WH 8501. ISME J. 4: 621–632. 20107492 10.1038/ismej.2009.148

[bib55] Siaut M. Cuine S. Cagnon C. Fessler B. Nguyen M., 2011 Oil accumulation in the model green alga *Chlamydomonas reinhardtii*: characterization, variability between common laboratory strains and relationship with starch reserves. BMC Biotechnol. 11: 7. 21255402 10.1186/1472-6750-11-7 PMC3036615

[bib56] Song Y. H. Ito S. Imaizumi T., 2013 Flowering time regulation: photoperiod- and temperature-sensing in leaves. Trends Plant Sci. 18: 575–583. 23790253 10.1016/j.tplants.2013.05.003 PMC3796012

[bib57] Stamatakis A., 2006 RAxML-VI-HPC: maximum likelihood-based phylogenetic analyses with thousands of taxa and mixed models. Bioinformatics 22: 2688–2690. 16928733 10.1093/bioinformatics/btl446

[bib58] Teramoto H. Nakamori A. Minagawa J. Ono T. A., 2002 Light-intensity-dependent expression of Lhc gene family encoding light-harvesting chlorophyll-a/b proteins of photosystem II in *Chlamydomonas reinhardtii*. Plant Physiol. 130: 325–333. 12226512 10.1104/pp.004622 PMC166565

[bib59] Trapnell C. Pachter L. Salzberg S. L., 2009 TopHat: discovering splice junctions with RNA-Seq. Bioinformatics 25: 1105–1111. 19289445 10.1093/bioinformatics/btp120 PMC2672628

[bib60] Trapnell C. Williams B. A. Pertea G. Mortazavi A. Kwan G., 2010 Transcript assembly and quantification by RNA-Seq reveals unannotated transcripts and isoform switching during cell differentiation. Nat. Biotechnol. 28: 511–515. 20436464 10.1038/nbt.1621 PMC3146043

[bib61] Ueda H. R. Hayashi S. Chen W. Sano M. Machida M., 2005 System-level identification of transcriptional circuits underlying mammalian circadian clocks. Nat. Genet. 37: 187–192. 15665827 10.1038/ng1504

[bib62] Voigt J. Munzner P., 1987 The chlamydomonas cell-cycle is regulated by a light dark-responsive cell-cycle switch. Planta 172: 463–472. 24226064 10.1007/BF00393861

[bib63] von Gromoff E. D. Schroda M. Oster U. Beck C. F., 2006 Identification of a plastid response element that acts as an enhancer within the Chlamydomonas HSP70A promoter. Nucleic Acids Res. 34: 4767–4779. 16971458 10.1093/nar/gkl602 PMC1635268

[bib64] Yanovsky M. J. Kay S. A., 2002 Molecular basis of seasonal time measurement in *Arabidopsis*. Nature 419: 308–312. 12239570 10.1038/nature00996

[bib65] Zou C. Lehti-Shiu M. D. Thomashow M. Shiu S. H., 2009 Evolution of stress-regulated gene expression in duplicate genes of *Arabidopsis thaliana*. PLoS Genet. 5: e1000581. 19649161 10.1371/journal.pgen.1000581 PMC2709438

[bib66] Zou C. Sun K. Mackaluso J. D. Seddon A. E. Jin R., 2011 *Cis*-regulatory code of stress-responsive transcription in *Arabidopsis thaliana*. Proc. Natl. Acad. Sci. USA 108: 14992–14997. 21849619 10.1073/pnas.1103202108 PMC3169165

