# Supplementary material for: Prevalence, Evolution, and cis-Regulation of Diel Transcription in Chlamydomonas reinhardtii
Source: G3 (Bethesda). 2014 Oct 28;4(12):2461–71. doi: 10.1534/g3.114.015032 (PMC4267941; doi:10.1534/g3.114.015032)
Supplement: Supporting Information [file supp_4_12_2461__index.html]

Prevalence, Evolution, and cis-Regulation of Diel Transcription in Chlamydomonas reinhardtii — Supporting Information 

# Prevalence, Evolution, and *cis*-Regulation of Diel Transcription in *Chlamydomonas reinhardtii*

## Supporting Information for Panchy *et al.*, 2014

**Files in this Data Supplement:**

- Supporting Information - Figures S1-S7, Tables S1-S6, and Files S1-S9 (PDF, 1 MB)
- Figure S1 - Expression profiles of cell cycle genes (MAT3, E2F, CDKA1, and CDKB1) in *C. reinhardtii* grown in TAP (Tris-Acetate-Phosphate) culture. (PDF, 179 KB)
- Figure S2 - Period, amplitude, and phase of cyclic expression amongst predictions made by COSPOT, DFT, and both methods combined. (PDF, 357 KB)
- Figure S3 - Most over- and under-enriched GO terms amongst phase clusters of cycling genes. (PDF, 309 KB)
- Figure S4 - Divergence of duplicate gene expression state modeled as a system of difference equations. (PDF, 192 KB)
- Figure S5 - Precision-recall and AUC-ROC curves of SVM predictions for *C. reinhardtii*. (PDF, 263 KB)
- Figure S6 - Regression of the AUC-ROC of phase-expression clusters against cluster size, and Pearson Correlation Coefficient (PCC) of genes in the cluster. (PDF, 362 KB)
- Figure S7 - Distribution of Fourier Transform cyclic score and COSPOT p-values. (PDF, 245 KB)
- Table S1 - Fisher's exact test p-values of GO terms with over-represented numbers of *C. reinhardtii* cycling genes. (PDF, 168 KB)
- Table S2 - Descriptions of the GO terms in each of the five broad functional categories. (PDF, 140 KB)
- Table S3 - Optimal parameters and performance measures of SVM classification. (PDF, 134 KB)
- Table S4 - "Gold Standard" cycling genes in *C. reinhardtii*. (PDF, 166 KB)
- Table S5 - Performance COSPOT and DFT on *C. reinhardtii*. (PDF, 164 KB)
- Table S6 - Performance of combining COPSOT and DFT on *C. reinhardtii*. (PDF, 165 KB)
- File S1 - Supplemental Materials and Methods (PDF, 147 KB)
- File S2 - Archive of gene trees in Nexus (.nex) format. (.zip, 2 MB)
- File S3 - Fisher's exact test p-values of GO terms with over-represented numbers of *C. reinhardtii* cycling genes in each phase cluster. (.xlsx, 31 KB)
- File S4 - Fisher's exact test p-values of GO terms with over-represented number of *A. thaliana* cycling genes in phase clusters. (.xlsx, 73 KB)
- File S5 - All motifs enriched in the promoters of cycling genes in *C. reinhardtii* clustered by phase. (.txt, 889 KB)
- File S6 - Phase, AUC-ROC, and composition of the best predicted expression clusters. (.xlsx, 13 KB)
- File S7 - List of motifs which were informative in predicting phase-expression clusters in TAMO format. (.txt, 64 KB)
- File S8 - Phase, AUC-ROC, and composition of the best predicted functional clusters. (.xlsx, 11 KB)
- File S9 - List of motifs which were informative in predicting phase-function clusters in TAMO format. (.txt, 21 KB)
