# Supplementary material for: Prevalence, Evolution, and cis-Regulation of Diel Transcription in Chlamydomonas reinhardtii
Source: G3 (Bethesda). 2014 Oct 28;4(12):2461–71. doi: 10.1534/g3.114.015032 (PMC4267941; doi:10.1534/g3.114.015032)
Supplement: Supporting Information [file supp_g3.114.015032_015032SI.pdf]

G3: Genes | Genomes | Genetics

Prevalence, evolution and *cis*-regulation of diel transcription in *Chlamydomonas reinhardtii*

Nicholas Panchy[*], Guangxi Wu[†], Linsey Newton[‡], Chia-Hong Tsai[‡,§], Jin Chen[*,§,**], Christoph Benning[††], Eva M. Farre[*,‡,1], Shin-Han Shiu[*, †,‡,1]

[*]Genetics Program, Michigan State University, East Lansing, MI 48824, USA

[†]Cellular and Molecular Biology Program, Michigan State University, East Lansing, MI 48824, USA

[‡]Department of Plant Biology, Michigan State University, East Lansing, MI 48824, USA

[§]MSU-DOE Plant Research Laboratory, Michigan State University, East Lansing, MI 48824, USA

[**]Department of Computer Science & Engineering, Michigan State University, East Lansing, MI 48824, USA

[††]Department of Biochemistry and Molecular Biology, Michigan State University, East Lansing, MI 48824, USA

DOI: 10.1534/g3.114.015032

File S1

SUPPLEMENTAL MATERIALS AND METHODS

Determining threshold scores for COSPOT and DFT

MITTAG *et al*. (2005) lists 18 proteins in *C. reinhardtii* which have previously show to exhibit circadian changes in the rate of transcription or concentration of mRNA. Amino acids sequences of these proteins were identified through KEGG and mapped to the *C. reinhardtii* genome using the TBLASTN tool available through Phytozome. We found fifteen proteins which mapped unambiguously to the *C. reinhardtii* genome and had matching annotation, only one of which was not present in the mRNA seq data set (**Table S4**).

To define a cutoff threshold for each of our methods, COSPOT and the DFT, each program was evaluated against our gold-standard set three different *p*-values thresholds (or the equivalent cyclic-score): 0.05, 0.02, and 0.01. For each *p*-value threshold, the coverage of both the gold-standard set and the whole *C. reinhardtii* genome is reported in **Table S5**. At each *p*-value threshold, the union of predictions from methods was used to define cyclically expressed genes in *C. reinhardtii*. As such, the *p*-value of the new two-dimensional threshold is defined by the joint distribution of COSPOT and DFT scores. Calculating this value is complicated by the fact that these scores are highly correlated ($R^2 > 0.7$), but the joint probability can be estimated using a randomized population of expression vectors (**Table S6**). For every test *p*-value threshold, the increase in joint probability (compared to individual significant thresholds) was relatively moderate whereas coverage of the gold standard increased by as much 20% over a single method. We chose to use the combination of COSPOT&DFT as our predictive method with a test *p*-value threshold of 0.02, which balances in the inflation of the joint probability with the coverage of the gold standard set.

The combined method is most effective at excluding non-cycling genes, rather than defining cycling genes, which can be seen by looking at the correlation of both methods at different scoring threshold. (**Figure S7**). While the overall correlation between both methods is high, the correlation amongst highly scoring genes (exceeding the 0.02 threshold for either method) is actually quite low ($R^2 < 0.2$). Genes which score very highly with one method may be at or just below the margin for the other, however, a gene which scores poorly in one method generally scores poorly with the other. Therefore, we chose a more conservative score threshold as a cautionary measure.

Derivation of the model of duplicate gene divergence

Divergence of expression state was modeled using the following system of difference equations:

$$C_{t+1} = C_t \cdot (1-d) + \frac{D_t s}{2} \qquad (3)$$

$$N_{t+1} = N_t \cdot (1-d) + \frac{D_t s}{2} \qquad (4)$$

$$D_{t+1} = D_t \cdot (1-s) + C_t d + N_t d \qquad (5)$$

Where C, N, and D represent the frequencies of cycling, non-cycling, and divergent duplicates at a given *Ks* (subscript “t”) and the subsequent *Ks* (subscript “t+1”). The variables d and s are, respectively, the probabilities of divergence from the identical state and reversion to the identical state. Since the null model assumes no bias, d and s are insensitive to whether the identical state is cycling or non-cycling.

Solving equations (3) and (4) for d, we obtain:

$$d = 1 + \frac{D_t s}{2C_t} - \frac{C_{t+1}}{C_t} \qquad (6)$$

$$d = 1 + \frac{D_t s}{2N_t} - \frac{N_{t+1}}{N_t} \qquad (7)$$

Using the property that the right hand sides of (6) and (7) must be equal, we arrive at the following formula for s that depends solely on duplicate frequencies:

$$s = \frac{\frac{2C_{t+1}}{C_t D_t} - \frac{2N_{t+1}}{N_t D_t}}{\frac{1}{C_t} - \frac{1}{N_t}} \qquad (8)$$

Initial conditions were set equal to values of C, D, and N observed at *Ks* = 0.3. We first attempted to fit values for d and s using frequencies at *Ks* 0.3 and 0.6, however because the percentage change in C is greater than N, we obtained a negative value for s. Since s is a probability, this results is unrealistic, so instead we fit d and s using *Ks* 0.6 and 0.9, obtaining values for d (0.42) and s (0.53) that were within [0,1]. Using these parameters, our model was able to replicate the overall behavior we observed, including the initial dip in C, though the percentage change is less than that of N (**Figure S3**). The root mean squared error between our predictions and observation was 0.03.

REFERENCES

MITTAG, M., S. KIAULEHN and C. H. JOHNSON, 2005 The circadian clock in Chlamydomonas reinhardtii. What is it for? What is it similar to? Plant Physiol 137: 399-40

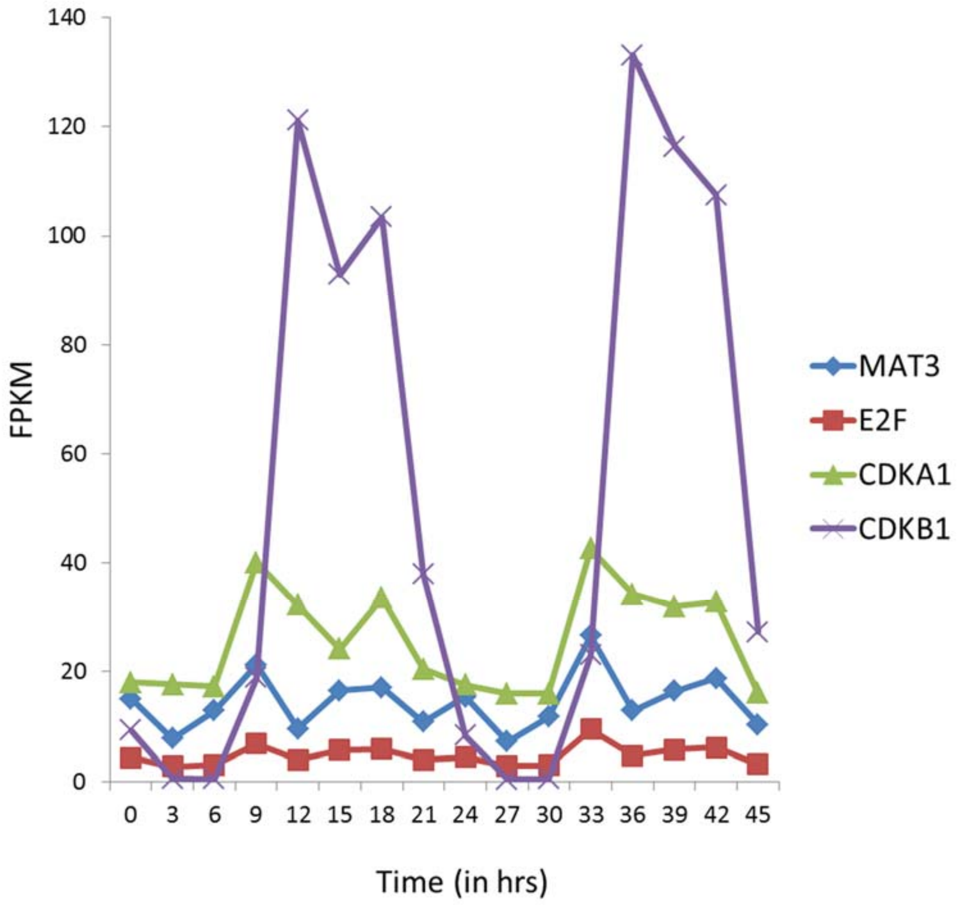

Figure S1 Expression profiles of cell cycle genes (MAT3, E2F, CDKA1, and CDKB1) in C. reinhardtii grown in TAP (Tris-Acetate-Phosphate) culture. As observed in previous studies of C. reinhardtii grown on autotrophic conditions (BISOVA et al. 2005), MAT3, CDKA1, and CDKB1 are most highly expressed between 12 and 18 hours after dawn, while E2F expression increases slightly earlier (between 6 and 9 hours).

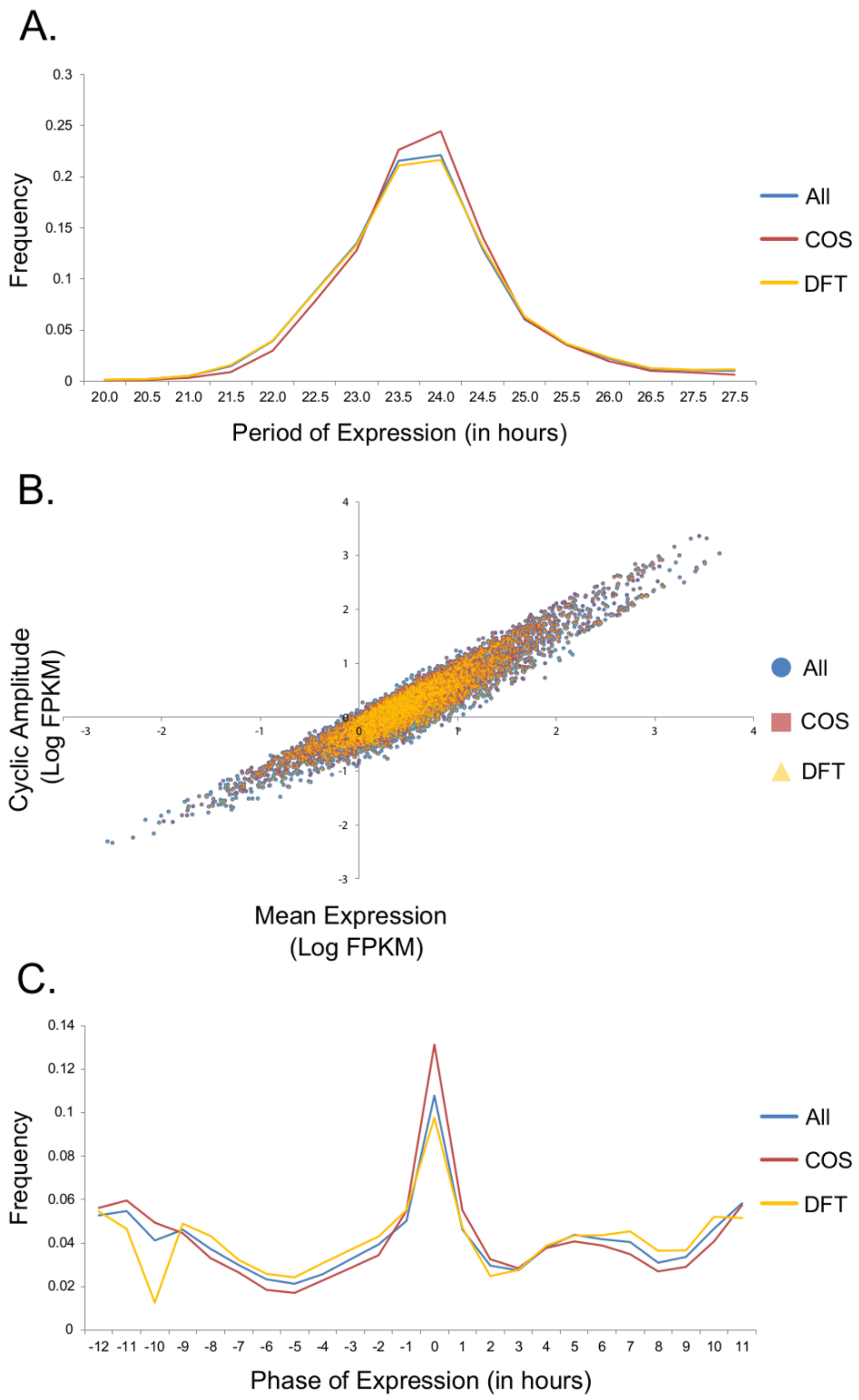

Figure S2 Period, amplitude, and phase of cyclic expression amongst predictions made by COSPOT, DFT, and both methods combined. (A) The distribution of the period of expression in cycling genes predicted by COSPOT (red), DFT (yellow) and both methods combined (blue). (B) Mean expression (x-axis) vs. the amplitude of cyclic expression (y-axis) of cycling genes. Color labels follow (A). (C) Phase of expression of cycling genes. Color labels follow (A).

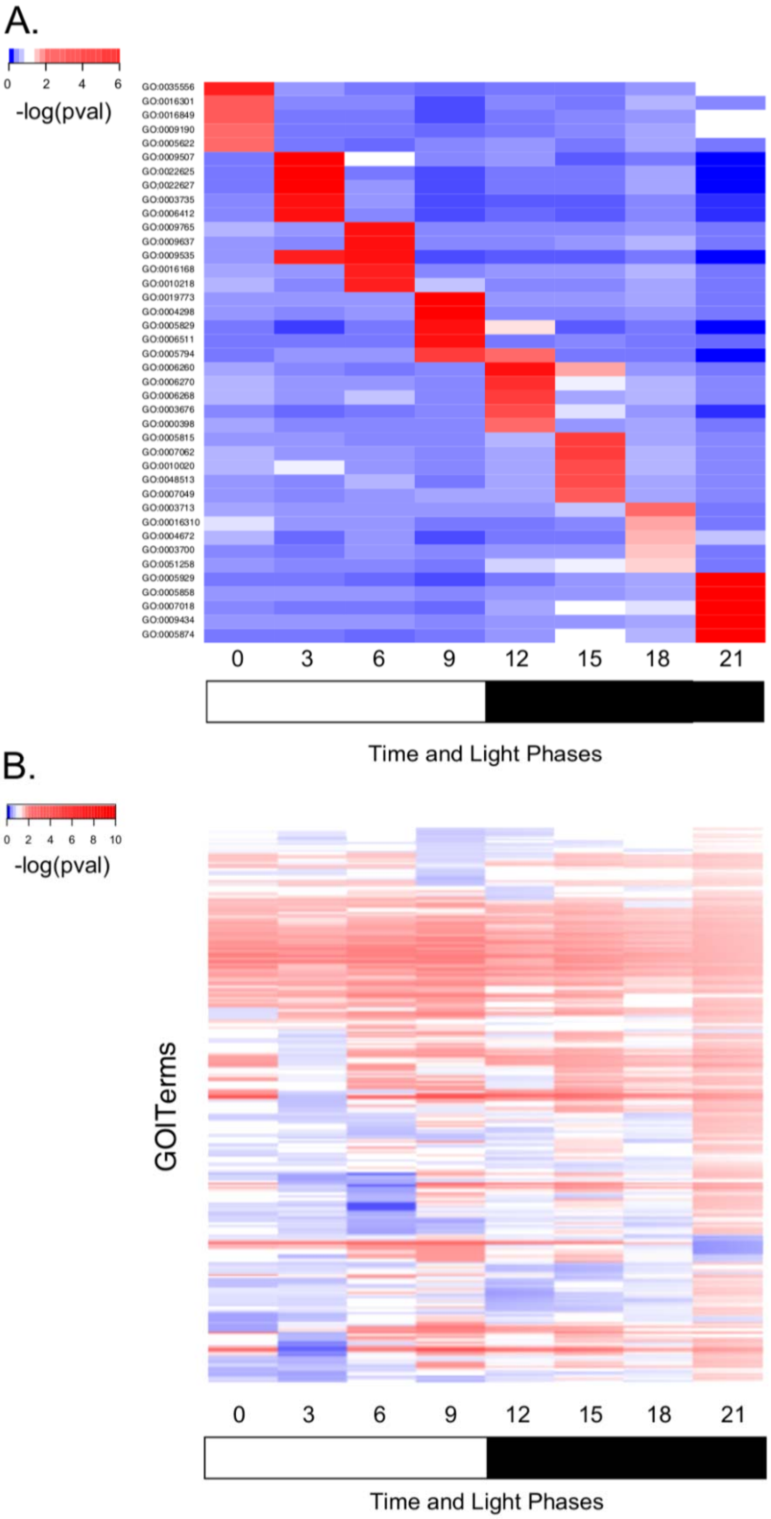

Figure S3 Most over- and under-enriched GO terms amongst phase clusters of cycling genes. (A) Heatmap showing the –log10 transformed Fisher's exact test *p*-values (pval) of the top five GO terms with over-represented numbers of genes in each phase cluster (ZT 0, 3, 6, 9, 12, 15, 18, and 21). (B) Heatmap showing transformed p-values of GO terms with under-represented numbers of genes in at least one phase cluster (same as in (A)). P-values were calculated and transformed as in part (A) except that the left-tail p-value was used.

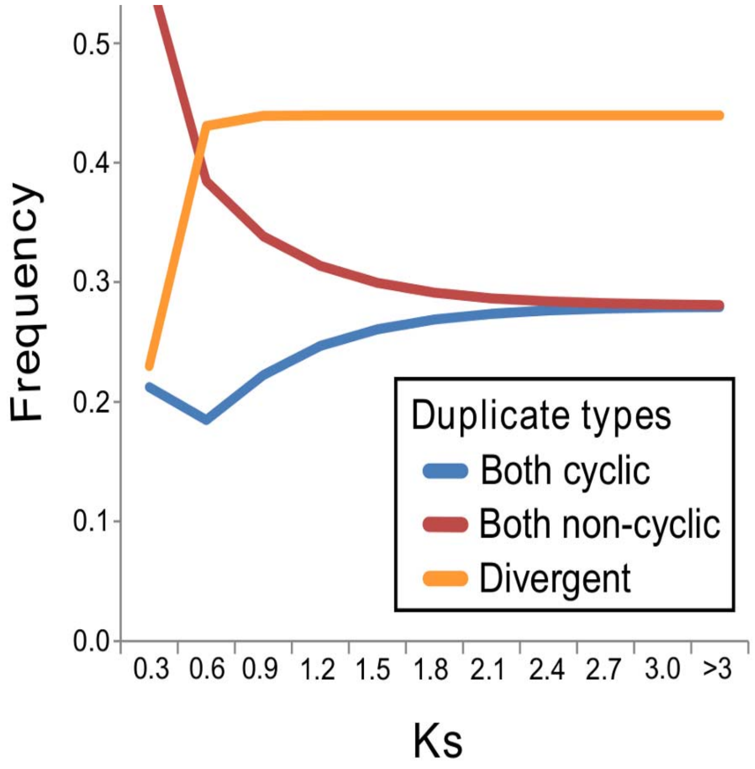

Figure S4 Divergence of duplicate gene expression state modeled as a system of difference equations. The frequency at which duplicate pairs in *C. reinhardtii* are both cycling (blue), both non-cycling (red), or divergent expression (orange) as a function of the synonymous substitution rate (*Ks*). The difference equations used to generate these data are described in the Supporting Information.

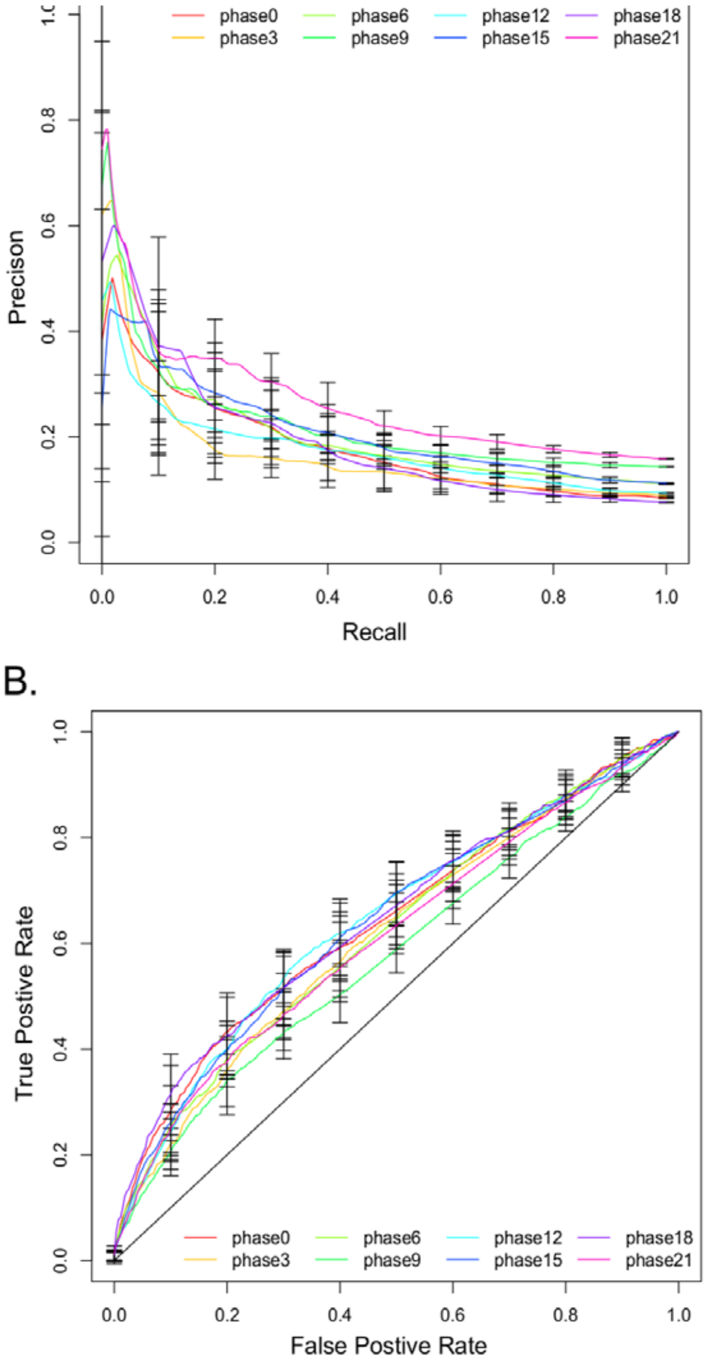

Figure S5 Precision-recall and AUC-ROC curves of SVM predictions for *C. reinhardtii*. (A) Precision-recall curves for the prediction of the each of the eight phase clusters in cycling genes in *C. reinhardtii* as classified using SVM. Each phase-cluster is represented as a different colored line: 0 (red), 3 (orange), 6 (lime), 9 (green), 12 (teal), 15 (blue), 18 (purple), 21 (pink). Error bars represent the variance in 10 separate runs of the SVM classifier at optimal parameters. (B) ROC curves for the prediction of each of the eight phase clusters in cycling genes in *C. reinhardtii* as classified using SVM. Line color and error bars are assigned as in (A).

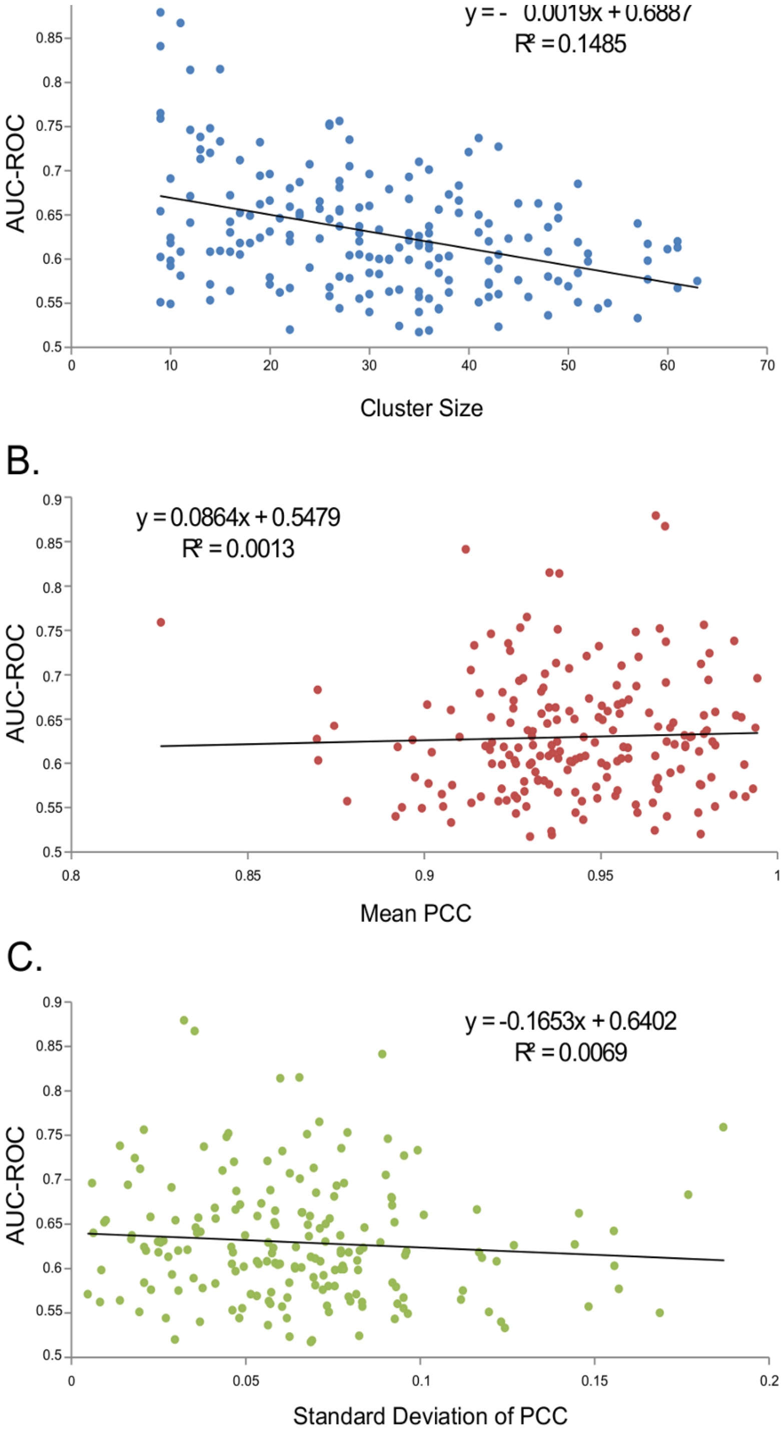

Figure S6 Regression of the AUC-ROC of phase-expression clusters against cluster size, and Pearson Correlation Coefficient (PCC) of genes in the cluster. (A) Plot of phase-expression cluster size against AUC-ROC. The black line indicates the best linear regression of AUC-ROC against cluster size. The equation is reported above the figure. (B) Plot of the mean PCC amongst genes in each phase-expression cluster against AUC-ROC. The black line indicates the best linear regression of AUC-ROC against mean PCC. The equation is reported above the figure. (C) Plot of the standard deviation of PCC amongst genes in each expression cluster against AUC-ROC. The black line indicates the best linear regression of AUC-ROC against standard deviation of PCC. The equation is reported above the figure.

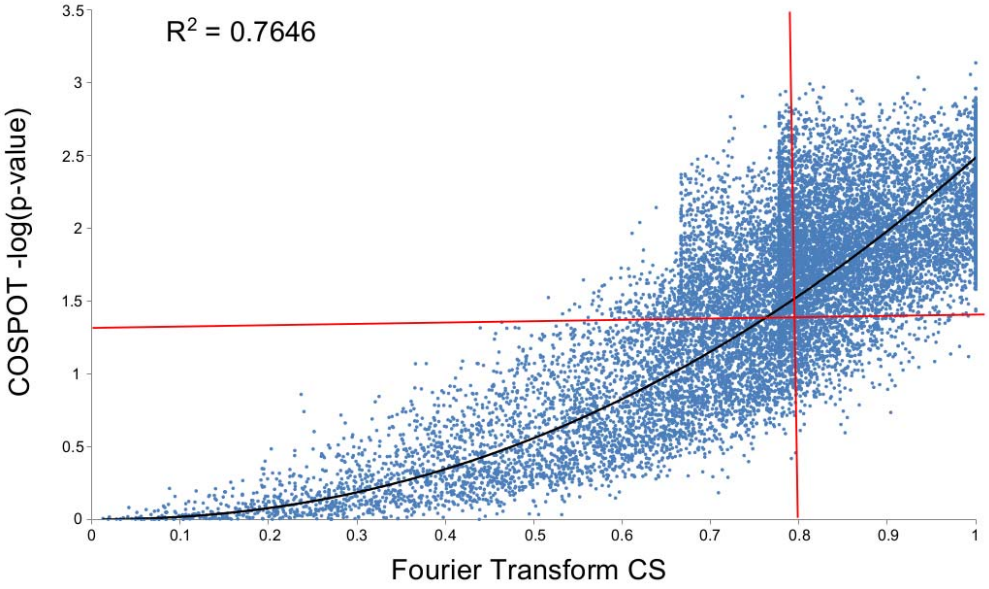

Figure S7 Distribution of Fourier Transform cyclic score and COSPOT p-values. Plot of Fourier Transform cyclic score (x-axis) against the negative log transform of the COSPOT *p*-value (y-axis). The black line is the best fit power-law regression of the transformed COSPOT *p*-value against Fourier Transform cyclic score. The red lines indicated the score threshold at a significance level of $\alpha < 0.02$ for the Fourier Transform cyclic score (vertical) and the transformed COSPOT *p*-value (horizontal).

Table S1 Fisher's exact test p-values of GO terms with over-represented numbers of *C. reinhardtii* cycling genes

GO Term	adjusted p-value[1]	Description
GO:0019861	4.38E-28	flagellum
GO:0005929	3.07E-11	cilium
GO:0035086	4.41E-06	axoneme
GO:0005874	1.16E-04	microtubule
GO:0007018	4.59E-04	microtubule-based movement
GO:0010287	4.59E-04	regulation of glucose transport
GO:0003777	6.53E-04	microtubule motor activity
GO:0009765	1.19E-03	carbohydrate mediated signaling
GO:0022625	1.19E-03	cytosolic large ribosomal subunit
GO:0006260	1.71E-03	DNA replication
GO:0030286	1.89E-03	dynein complex
GO:0005886	2.16E-03	plasma membrane
GO:0030030	6.12E-03	cell projection organization
GO:0005794	6.52E-03	Golgi apparatus
GO:0042995	1.00E-02	cell projection
GO:0005198	1.00E-02	structural molecule activity
GO:0003774	1.00E-02	motor activity
GO:0022627	1.00E-02	cytosolic small ribosomal subunit
GO:0005774	1.42E-02	vacuolar membrane
GO:0009653	1.77E-02	anatomical structure morphogenesis
GO:0009535	1.77E-02	chloroplast thylakoid membrane
GO:0009637	2.20E-02	response to blue light
GO:0006364	2.20E-02	rRNA processing
GO:0005932	2.65E-02	microtubule basal body
GO:0009506	2.65E-02	plasmodesmata
GO:0004674	2.65E-02	protein serine/threonine kinase
GO:0005509	2.66E-02	calcium ion binding
GO:0005488	2.75E-02	binding
GO:0030992	2.75E-02	intraciliary transport particle B
GO:0009507	3.12E-02	chloroplast
GO:0010114	3.34E-02	response to red light
GO:0010218	3.34E-02	response to far red light
GO:0046686	3.79E-02	response to cadmium ion
GO:0009523	4.16E-02	photosystem II
GO:0048046	4.21E-02	apoplast
GO:0006270	4.21E-02	DNA replication initiation
GO:0009296	4.21E-02	flagellum assembly
GO:0010020	4.21E-02	chloroplast fission
GO:0009434	4.21E-02	motile cilium

GO:0044430	4.21E-02	cytoskeletal part
GO:0019253	4.21E-02	reductive pentose-phosphate cycle
GO:0019773	4.21E-02	proteasome core complex, alpha-subunit complex
GO:0009826	4.21E-02	unidimensional cell growth
GO:0004298	4.33E-02	threonine-type endopeptidase activity

1. Fisher Exact Test *p*-value adjusted according to Benjamini-Hochberg

Table S2 Descriptions of the GO terms in each of the five broad functional categories

Category	GO Terms	Description
**photosynthesis and light response**	GO:0015671	oxygen transport
	GO:0009773	photosynthetic electron transport in photosystem I
	GO:0010206	photosystem II repair
	GO:0009765	photosynthesis, light harvesting
	GO:0015979	photosynthesis
	GO:0010218	response to far red light
	GO:0009637	response to blue light
	GO:0010114	response to red light
	GO:0010304	PSII associated light-harvesting complex II catabolic process
	GO:0010020	chloroplast fission
	GO:0009507	chloroplast
	GO:0009579	thylakoid
	GO:0009570	chloroplast stroma
	GO:0009941	chloroplast envelope
	GO:0009543	chloroplast thylakoid lumen
	GO:0009523	photosystem II
	GO:0009522	photosystem I
	GO:0009533	chloroplast stromal thylakoid
	GO:0009534	chloroplast thylakoid
	GO:0010287	plastoglobule
	GO:0009535	chloroplast thylakoid membrane
	GO:0016168	chlorophyll binding
**cell cycle and mitosis**	GO:0006260	DNA replication
	GO:0006270	DNA replication initiation
	GO:0006268	DNA unwinding involved in replication
	GO:0000910	cytokinesis
	GO:0000724	double-strand break repair via homologous recombination
	GO:0006302	double-strand break repair
	GO:0007062	sister chromatid cohesion
	GO:0007067	mitosis
	GO:0006259	DNA metabolic process
	GO:0007049	cell cycle
	GO:0051726	regulation of cell cycle

	GO:0006281	DNA repair
	GO:0051301	cell division
	GO:0006310	DNA recombination
	GO:0005819	spindle
	GO:0005815	microtubule organizing center
	GO:0005694	chromosome
	GO:0004003	ATP-dependent DNA helicase activity
	GO:0003887	DNA-directed DNA polymerase activity
	GO:0004386	helicase activity
**microtubules and flagella**	GO:0000226	microtubule cytoskeleton organization
	GO:0007018	microtubule-based movement
	GO:0009296	flagellum assembly
	GO:0030030	cell projection organization
	GO:0042384	cilium assembly
	GO:0015630	microtubule cytoskeleton
	GO:0044430	cytoskeletal part
	GO:0019861	flagellum
	GO:0030286	dynein complex
	GO:0035086	cilium axoneme
	GO:0005813	centrosome
	GO:0005932	microtubule basal body
	GO:0005874	microtubule
	GO:0005929	cilium
	GO:0005856	cytoskeleton
	GO:0005858	axonemal dynein complex
	GO:0035085	cilium axoneme
	GO:0009434	motile cilium
	GO:0030992	intraflagellar transport particle B
	GO:0042995	cell projection
	GO:0044463	cell projection part
	GO:0005876	spindle microtubule
	GO:0003777	microtubule motor activity
	GO:0003774	motor activity
	GO:0004835	tubulin-tyrosine ligase activity
**mitochondria and metabolism**	GO:0006096	glycolysis
	GO:0006122	mitochondrial electron transport, ubiquinol to cytochrome c
	GO:0005983	starch catabolic process
	GO:0006098	pentose-phosphate shunt
	GO:0007005	mitochondrion organization

	GO:0006508	proteolysis
	GO:0015986	ATP synthesis coupled proton transport
	GO:0045261	proton-transporting ATP synthase complex, catalytic core F(1)
	GO:0005750	mitochondrial respiratory chain complex III
	GO:0005747	mitochondrial respiratory chain complex I
	GO:0005739	mitochondrion
	GO:0005759	mitochondrial matrix
	GO:0005741	mitochondrial outer membrane
	GO:0005743	mitochondrial inner membrane
	GO:0046933	proton-transporting ATP synthase activity, rotational mechanism
	GO:0046961	proton-transporting ATPase activity, rotational mechanism
**ribosome and translation**	GO:0006414	translational elongation
	GO:0006412	translation
	GO:0022626	cytosolic ribosome
	GO:0022625	cytosolic large ribosomal subunit
	GO:0022627	cytosolic small ribosomal subunit
	GO:0019843	rRNA binding
	GO:0003735	structural constituent of ribosome

Table S3 Optimal parameters and performance measures of SVM classification

Phase	C[1]	R[2]	AUC-ROC	F-measure	Precision	Recall
0	0.01	4	0.64	0.22	0.24	0.20
3	0.1	1.5	0.62	0.21	0.14	0.39
6	0.1	4	0.62	0.19	0.26	0.15
9	0.1	2.5	0.58	0.22	0.27	0.18
12	0.01	3.5	0.64	0.19	0.21	0.18
15	0.1	1.5	0.64	0.27	0.21	0.40
18	0.1	4	0.65	0.23	0.24	0.23
21	0.01	3.5	0.61	0.21	0.38	0.15

1. C = minimum separation

2. R = ratio of negative to positive examples

Table S4 "Gold Standard" cycling genes in *C. reinhardtii*

Gene	Name	Reference	Locus	DFT Cyclic Score	COSPOT p-value
ATP2/ARF1	ADP-ribosylation factor	MEMON *et al.* (1995)	Cre17.g698000	0.90	1.9e-02
CAH1	carbonic anhydrase	FUJIWARA *et al.* (1996)	Cre04.g223100	0.76	1.7e-01
CYC4	cytochrome c	JACOBSHAGEN *et al.* (2001)	Cre16.g670950	0.78	6.2e-01
Cytosolic thioredoxin h1	Cytosolic thioredoxin h1	LEMAIRE *et al.* (1999)	Cre09.g391900	0.29	4.0e-01
FBA1	chloroplastic fructose-bisphosphate aldolase	JACOBSHAGEN *et al.* (2001)	Cre01.g006950	0.95	1.2e-02
FBA2	chloroplastic fructose-bisphosphate aldolase	JACOBSHAGEN *et al.* (2001)	Cre02.g093450	0.83	3.4e-02
FBA3	chloroplastic fructose-bisphosphate aldolase	JACOBSHAGEN *et al.* (2001)	Cre05.g234550	0.9	2.3e-02
FBA4	chloroplastic fructose-bisphosphate aldolase	JACOBSHAGEN *et al.* (2001)	Cre02.g115650	0.61	1.9e-02
FNR1	Ferredoxin NADP reductase	LEMAIRE *et al.* (1999)	Cre11.g476750	0.81	2.4e-02
HSP70B	70kd family heat shock protein	JACOBSHAGEN *et al.* (2001)	Cre06.250100	0.32	8.5e-01
LCHII	Chlorophyll binding protein	JACOBSHAGEN *et al.* (1996)	Cre06.g283950	0.82	6.5e-03
PRK1	phosphoribulokinase	LEMAIRE *et al.* (1999)	Cre12.g554800	0.99	1.0e-02
TUB1	Beta-tubulin	JACOBSHAGEN & JOHNSON (1994)	Cre12.g542250	0.67	1.1e-02
TUB2	Beta-tubulin	JACOBSHAGEN & JOHNSON (1994)	Cre12.g549550	0.98	1.1e-02
TufA	Elongation factor Tu	HWANG *et al.* (1996)	Cre06.g259150	0.77	1.1e-02

Table S5 Performance COSPOT and DFT on *C. reinhardtii*

Method and α levels	Genome Coverage[1]	Gold Stand Coverage[2]
**COSPOT**		
α = 0.01	21.0% (3590)	6.7% (1)
α = 0.02	37.4% (6400)	46.7% (7)
α = 0.05	54.9% (9392)	73.3% (11)
**DFT**		
α = 0.01	29.6% (5061)	33.3% (5)
α = 0.02	37.6% (6443)	53.3% (8)
α = 0.05	55.8% (9556)	73.3% (11)

1. Parentheses indicated the actual number of genes covered

2. Parentheses indicated how many of 15 gold standard genes are identified as cyclic

Table S6 Performance of combining COPSOT and DFT on *C. reinhardtii*

Test P-value	Joint Probability[1]	Overlap[2]	Genome Coverage[3]	Gold Stand Coverage[4]
α = 0.01	0.0134	39.6% (2414)	35.7% (6236)	40.0% (6)
α = 0.02	0.0272	56.7% (4579)	47.2% (8072)	73.3% (11)
α = 0.05	0.0734	73.5% (8024)	61.7% (10552)	86.6% (13)

1. The joint probability of a gene having a score with a p-value of α in either COSPOT or DFT
2. Parentheses indicated the actual number of genes in the overlap set
3. Parentheses indicated the actual number of genes covered
4. Parentheses indicated how many of 15 gold standard genes are identified as cyclic

Files S2-S9

Available for download at http://www.g3journal.org/lookup/suppl/doi:10.1534/g3.114.015032/-/DC1

File S2 Archive of gene trees in Nexus (.nex) format

File S3 Fisher's exact test p-values of GO terms with over-represented numbers of *C. reinhardtii* cycling genes in each phase cluster

File S4 Fisher's exact test p-values of GO terms with over-represented number of *A. thaliana* cycling genes in phase clusters

File S5 All motifs enriched in the promoters of cycling genes in *C. reinhardtii* clustered by phase

File S6 Phase, AUC-ROC, and composition of the best predicted expression clusters

File S7 List of motifs which were informative in predicting phase-expression clusters in TAMO format

File S8 Phase, AUC-ROC, and composition of the best predicted functional clusters

File S9 List of motifs which were informative in predicting phase-function clusters in TAMO format
