# Supplementary material for: Prevalence, Evolution, and cis-Regulation of Diel Transcription in Chlamydomonas reinhardtii
Source: G3 (Bethesda). 2014 Oct 28;4(12):2461–71. doi: 10.1534/g3.114.015032 (PMC4267941; doi:10.1534/g3.114.015032)
Supplement: Supporting Information [file supp_g3.114.015032_FigureS1.pdf]

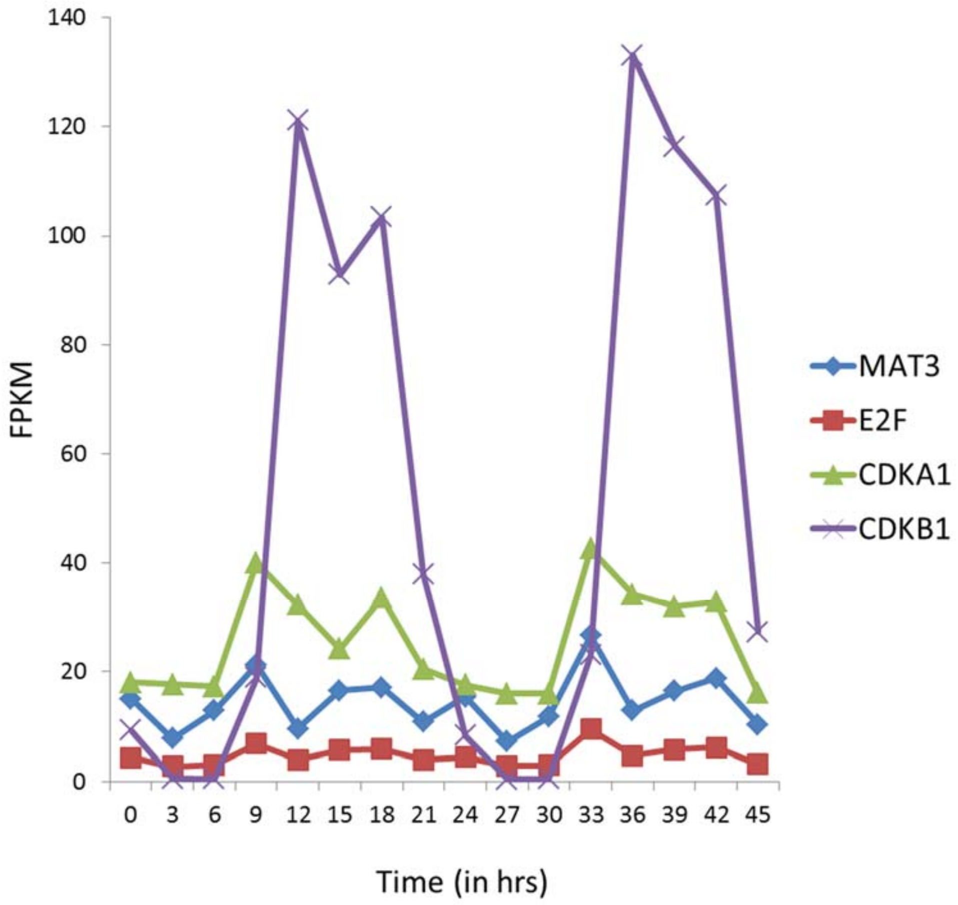

Figure S1 Expression profiles of cell cycle genes (MAT3, E2F, CDKA1, and CDKB1) in C. reinhardtii grown in TAP (Tris-Acetate-Phosphate) culture. As observed in previous studies of C. reinhardtii grown on autotrophic conditions (BISOVA et al. 2005), MAT3, CDKA1, and CDKB1 are most highly expressed between 12 and 18 hours after dawn, while E2F expression increases slightly earlier (between 6 and 9 hours).
