# Supplementary material for: Prevalence, Evolution, and cis-Regulation of Diel Transcription in Chlamydomonas reinhardtii
Source: G3 (Bethesda). 2014 Oct 28;4(12):2461–71. doi: 10.1534/g3.114.015032 (PMC4267941; doi:10.1534/g3.114.015032)
Supplement: Supporting Information [file supp_g3.114.015032_FigureS2.pdf]

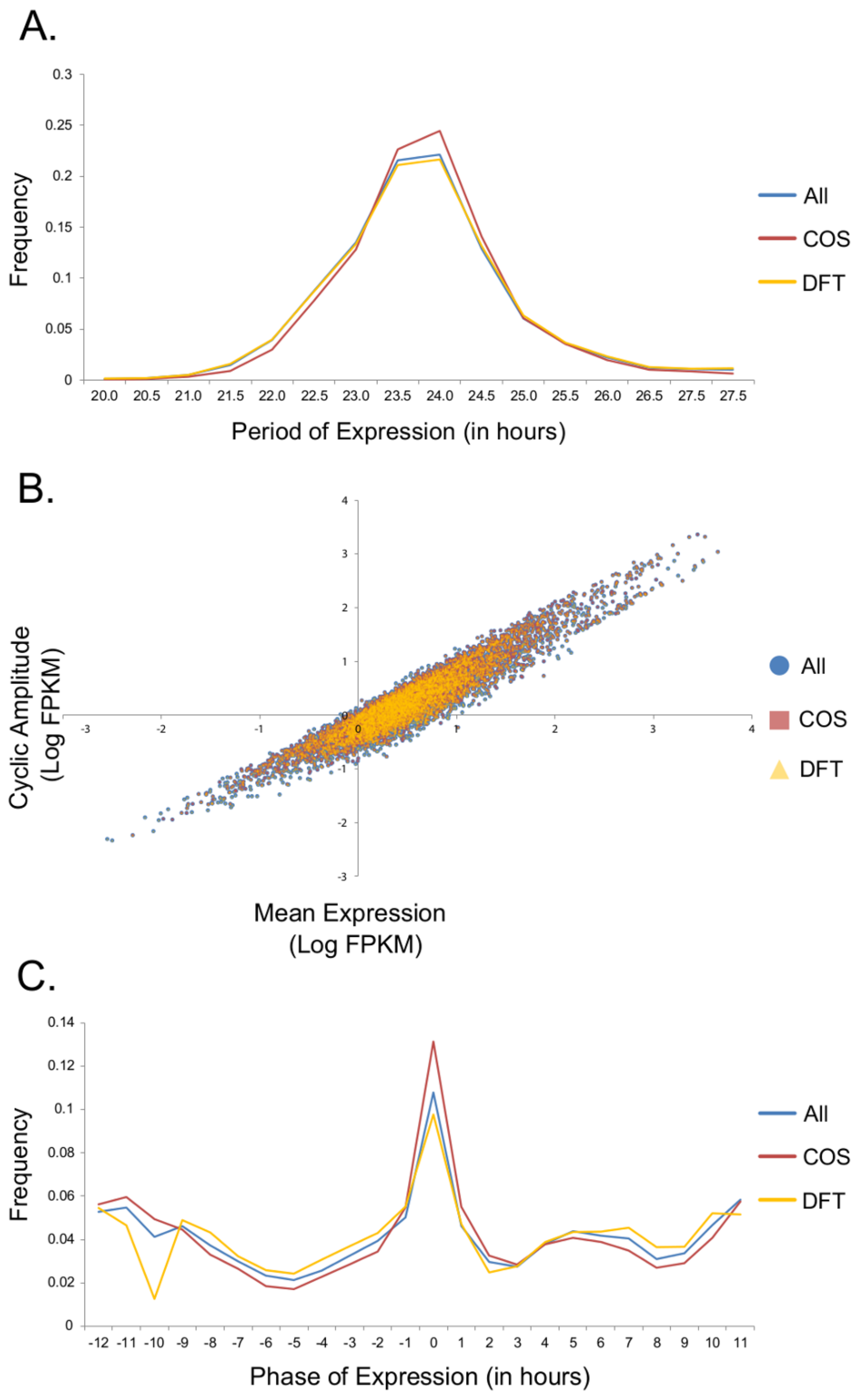

Figure S2 Period, amplitude, and phase of cyclic expression amongst predictions made by COSPOT, DFT, and both methods combined. (A) The distribution of the period of expression in cycling genes predicted by COSPOT (red), DFT (yellow) and both methods combined (blue). (B) Mean expression (x-axis) vs. the amplitude of cyclic expression (y-axis) of cycling genes. Color labels follow (A). (C) Phase of expression of cycling genes. Color labels follow (A).
