# Supplementary material for: Prevalence, Evolution, and cis-Regulation of Diel Transcription in Chlamydomonas reinhardtii
Source: G3 (Bethesda). 2014 Oct 28;4(12):2461–71. doi: 10.1534/g3.114.015032 (PMC4267941; doi:10.1534/g3.114.015032)
Supplement: Supporting Information [file supp_g3.114.015032_FigureS3.pdf]

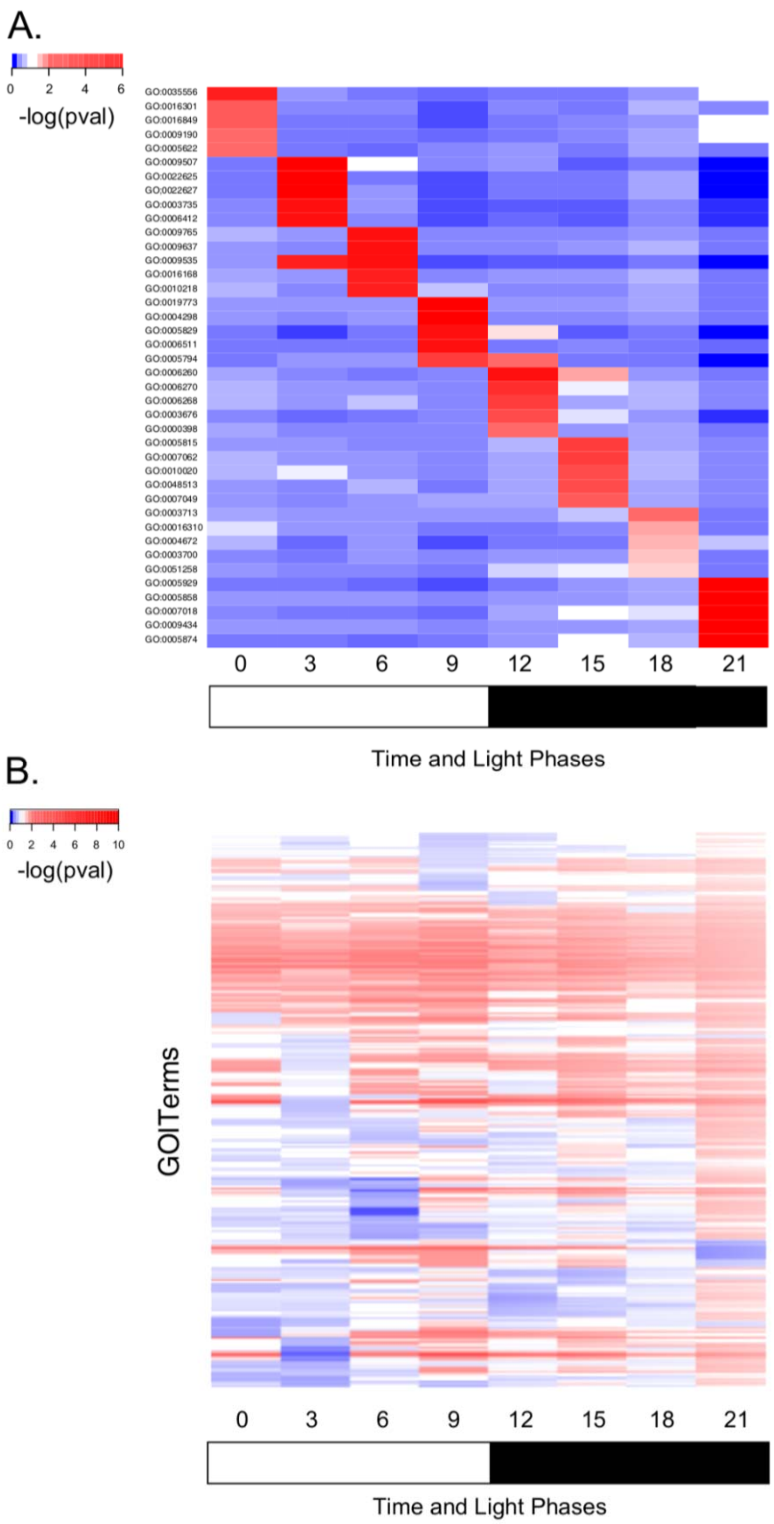

Figure S3 Most over- and under-enriched GO terms amongst phase clusters of cycling genes. (A) Heatmap showing the –log10 transformed Fisher's exact test *p*-values (pval) of the top five GO terms with over-represented numbers of genes in each phase cluster (ZT 0, 3, 6, 9, 12, 15, 18, and 21). (B) Heatmap showing transformed p-values of GO terms with under-represented numbers of genes in at least one phase cluster (same as in (A)). P-values were calculated and transformed as in part (A) except that the left-tail p-value was used.
