# Supplementary material for: Prevalence, Evolution, and cis-Regulation of Diel Transcription in Chlamydomonas reinhardtii
Source: G3 (Bethesda). 2014 Oct 28;4(12):2461–71. doi: 10.1534/g3.114.015032 (PMC4267941; doi:10.1534/g3.114.015032)
Supplement: Supporting Information [file supp_g3.114.015032_FigureS4.pdf]

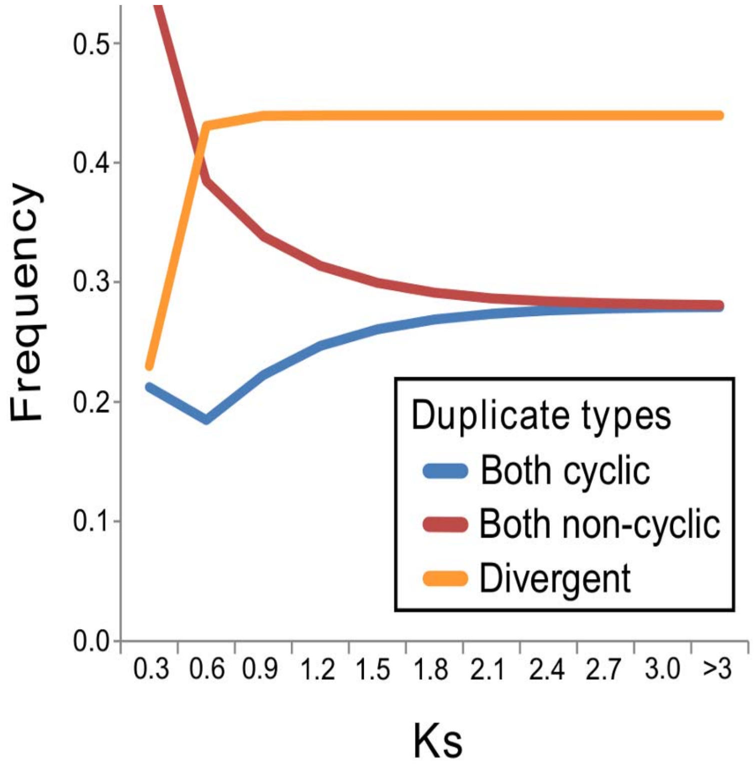

Figure S4 Divergence of duplicate gene expression state modeled as a system of difference equations. The frequency at which duplicate pairs in *C. reinhardtii* are both cycling (blue), both non-cycling (red), or divergent expression (orange) as a function of the synonymous substitution rate (*Ks*). The difference equations used to generate these data are described in the Supporting Information.
