# Supplementary material for: Prevalence, Evolution, and cis-Regulation of Diel Transcription in Chlamydomonas reinhardtii
Source: G3 (Bethesda). 2014 Oct 28;4(12):2461–71. doi: 10.1534/g3.114.015032 (PMC4267941; doi:10.1534/g3.114.015032)
Supplement: Supporting Information [file supp_g3.114.015032_FigureS5.pdf]

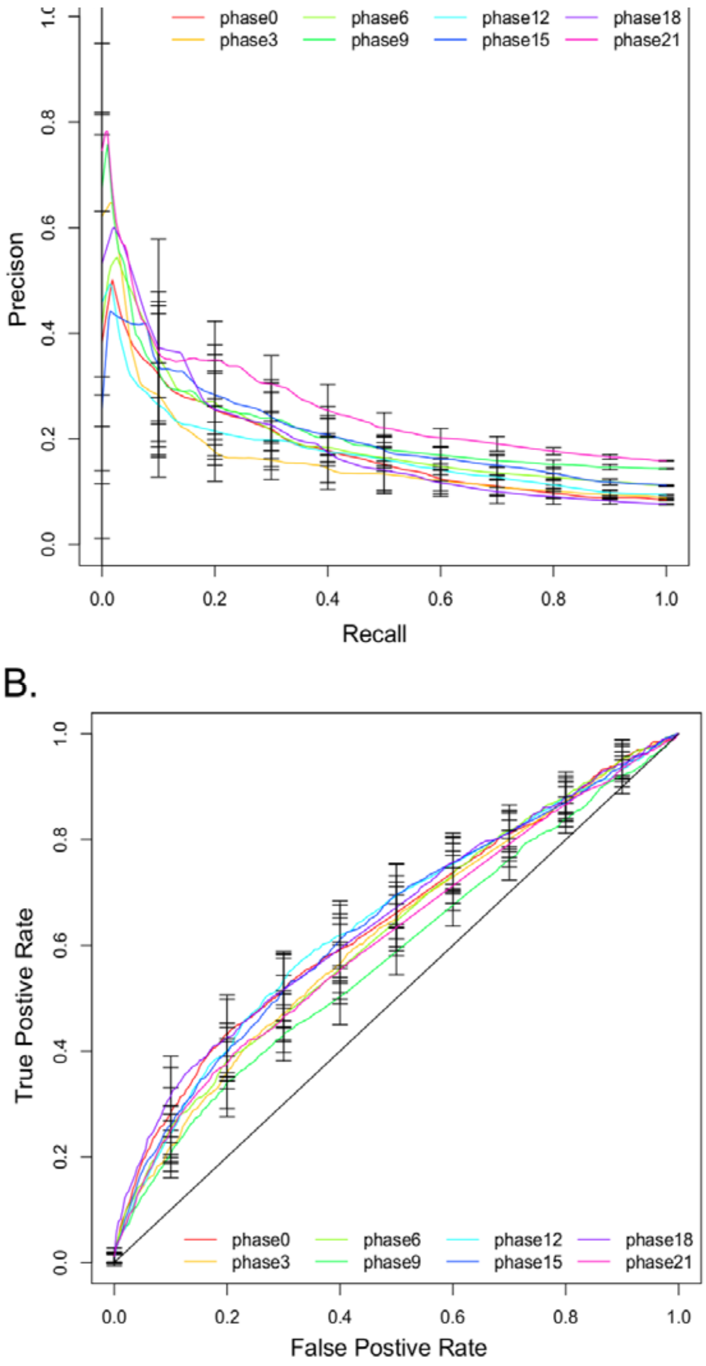

Figure S5 Precision-recall and AUC-ROC curves of SVM predictions for *C. reinhardtii*. (A) Precision-recall curves for the prediction of the each of the eight phase clusters in cycling genes in *C. reinhardtii* as classified using SVM. Each phase-cluster is represented as a different colored line: 0 (red), 3 (orange), 6 (lime), 9 (green), 12 (teal), 15 (blue), 18 (purple), 21 (pink). Error bars represent the variance in 10 separate runs of the SVM classifier at optimal parameters. (B) ROC curves for the prediction of each of the eight phase clusters in cycling genes in *C. reinhardtii* as classified using SVM. Line color and error bars are assigned as in (A).
