# Supplementary material for: Prevalence, Evolution, and cis-Regulation of Diel Transcription in Chlamydomonas reinhardtii
Source: G3 (Bethesda). 2014 Oct 28;4(12):2461–71. doi: 10.1534/g3.114.015032 (PMC4267941; doi:10.1534/g3.114.015032)
Supplement: Supporting Information [file supp_g3.114.015032_FigureS6.pdf]

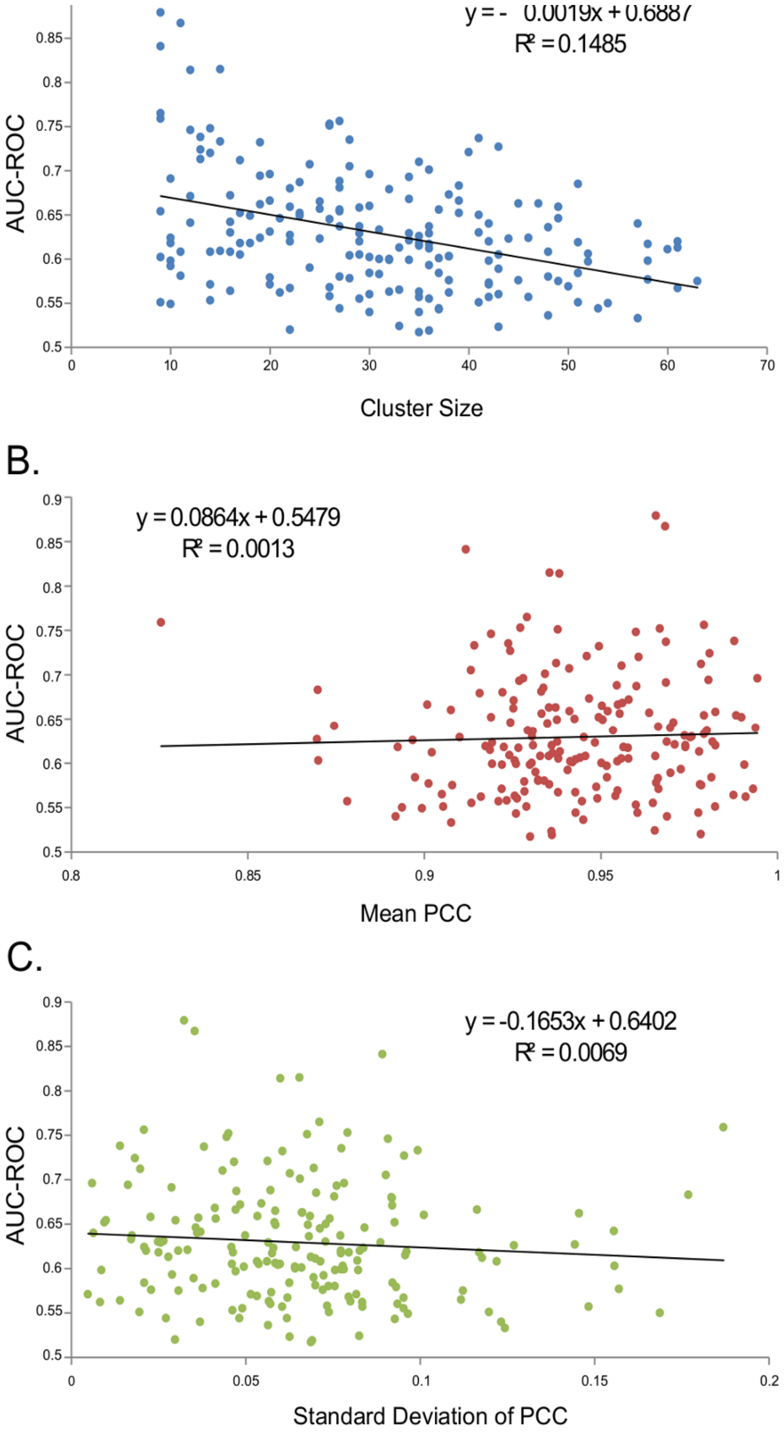

Figure S6 Regression of the AUC-ROC of phase-expression clusters against cluster size, and Pearson Correlation Coefficient (PCC) of genes in the cluster. (A) Plot of phase-expression cluster size against AUC-ROC. The black line indicates the best linear regression of AUC-ROC against cluster size. The equation is reported above the figure. (B) Plot of the mean PCC amongst genes in each phase-expression cluster against AUC-ROC. The black line indicates the best linear regression of AUC-ROC against mean PCC. The equation is reported above the figure. (C) Plot of the standard deviation of PCC amongst genes in each expression cluster against AUC-ROC. The black line indicates the best linear regression of AUC-ROC against standard deviation of PCC. The equation is reported above the figure.
