# Supplementary material for: Prevalence, Evolution, and cis-Regulation of Diel Transcription in Chlamydomonas reinhardtii
Source: G3 (Bethesda). 2014 Oct 28;4(12):2461–71. doi: 10.1534/g3.114.015032 (PMC4267941; doi:10.1534/g3.114.015032)
Supplement: Supporting Information [file supp_g3.114.015032_FigureS7.pdf]

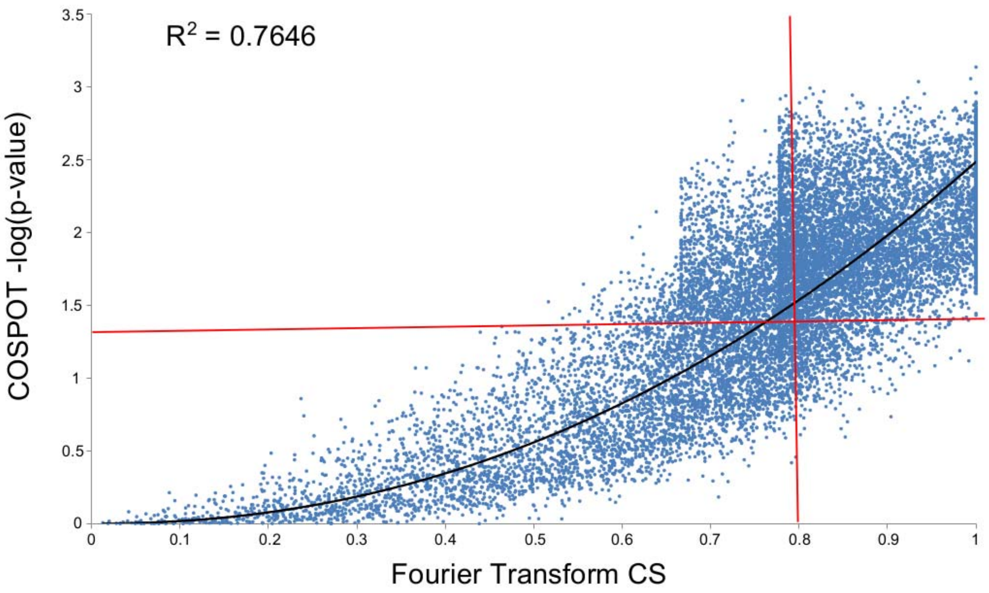

Figure S7 Distribution of Fourier Transform cyclic score and COSPOT p-values. Plot of Fourier Transform cyclic score (x-axis) against the negative log transform of the COSPOT *p*-value (y-axis). The black line is the best fit power-law regression of the transformed COSPOT *p*-value against Fourier Transform cyclic score. The red lines indicated the score threshold at a significance level of $\alpha < 0.02$ for the Fourier Transform cyclic score (vertical) and the transformed COSPOT *p*-value (horizontal).
