# Supplementary material for: Prevalence, Evolution, and cis-Regulation of Diel Transcription in Chlamydomonas reinhardtii
Source: G3 (Bethesda). 2014 Oct 28;4(12):2461–71. doi: 10.1534/g3.114.015032 (PMC4267941; doi:10.1534/g3.114.015032)
Supplement: Supporting Information [file supp_g3.114.015032_TableS1.pdf]

Table S1 Fisher's exact test p-values of GO terms with over-represented numbers of *C. reinhardtii* cycling genes

GO Term	adjusted p-value[1]	Description
GO:0019861	4.38E-28	flagellum
GO:0005929	3.07E-11	cilium
GO:0035086	4.41E-06	axoneme
GO:0005874	1.16E-04	microtubule
GO:0007018	4.59E-04	microtubule-based movement
GO:0010287	4.59E-04	regulation of glucose transport
GO:0003777	6.53E-04	microtubule motor activity
GO:0009765	1.19E-03	carbohydrate mediated signaling
GO:0022625	1.19E-03	cytosolic large ribosomal subunit
GO:0006260	1.71E-03	DNA replication
GO:0030286	1.89E-03	dynein complex
GO:0005886	2.16E-03	plasma membrane
GO:0030030	6.12E-03	cell projection organization
GO:0005794	6.52E-03	Golgi apparatus
GO:0042995	1.00E-02	cell projection
GO:0005198	1.00E-02	structural molecule activity
GO:0003774	1.00E-02	motor activity
GO:0022627	1.00E-02	cytosolic small ribosomal subunit
GO:0005774	1.42E-02	vacuolar membrane
GO:0009653	1.77E-02	anatomical structure morphogenesis
GO:0009535	1.77E-02	chloroplast thylakoid membrane
GO:0009637	2.20E-02	response to blue light
GO:0006364	2.20E-02	rRNA processing
GO:0005932	2.65E-02	microtubule basal body
GO:0009506	2.65E-02	plasmodesmata
GO:0004674	2.65E-02	protein serine/threonine kinase
GO:0005509	2.66E-02	calcium ion binding
GO:0005488	2.75E-02	binding
GO:0030992	2.75E-02	intraciliary transport particle B
GO:0009507	3.12E-02	chloroplast
GO:0010114	3.34E-02	response to red light
GO:0010218	3.34E-02	response to far red light
GO:0046686	3.79E-02	response to cadmium ion
GO:0009523	4.16E-02	photosystem II
GO:0048046	4.21E-02	apoplast
GO:0006270	4.21E-02	DNA replication initiation
GO:0009296	4.21E-02	flagellum assembly
GO:0010020	4.21E-02	chloroplast fission
GO:0009434	4.21E-02	motile cilium

GO:0044430	4.21E-02	cytoskeletal part
GO:0019253	4.21E-02	reductive pentose-phosphate cycle
GO:0019773	4.21E-02	proteasome core complex, alpha-subunit complex
GO:0009826	4.21E-02	unidimensional cell growth
GO:0004298	4.33E-02	threonine-type endopeptidase activity

1. Fisher Exact Test *p*-value adjusted according to Benjamini-Hochberg
