# Supplementary material for: Prevalence, Evolution, and cis-Regulation of Diel Transcription in Chlamydomonas reinhardtii
Source: G3 (Bethesda). 2014 Oct 28;4(12):2461–71. doi: 10.1534/g3.114.015032 (PMC4267941; doi:10.1534/g3.114.015032)
Supplement: Supporting Information [file supp_g3.114.015032_TableS2.pdf]

Table S2 Descriptions of the GO terms in each of the five broad functional categories

Category	GO Terms	Description
**photosynthesis and light response**	GO:0015671	oxygen transport
	GO:0009773	photosynthetic electron transport in photosystem I
	GO:0010206	photosystem II repair
	GO:0009765	photosynthesis, light harvesting
	GO:0015979	photosynthesis
	GO:0010218	response to far red light
	GO:0009637	response to blue light
	GO:0010114	response to red light
	GO:0010304	PSII associated light-harvesting complex II catabolic process
	GO:0010020	chloroplast fission
	GO:0009507	chloroplast
	GO:0009579	thylakoid
	GO:0009570	chloroplast stroma
	GO:0009941	chloroplast envelope
	GO:0009543	chloroplast thylakoid lumen
	GO:0009523	photosystem II
	GO:0009522	photosystem I
	GO:0009533	chloroplast stromal thylakoid
	GO:0009534	chloroplast thylakoid
	GO:0010287	plastoglobule
	GO:0009535	chloroplast thylakoid membrane
	GO:0016168	chlorophyll binding
**cell cycle and mitosis**	GO:0006260	DNA replication
	GO:0006270	DNA replication initiation
	GO:0006268	DNA unwinding involved in replication
	GO:0000910	cytokinesis
	GO:0000724	double-strand break repair via homologous recombination
	GO:0006302	double-strand break repair
	GO:0007062	sister chromatid cohesion
	GO:0007067	mitosis
	GO:0006259	DNA metabolic process
	GO:0007049	cell cycle
	GO:0051726	regulation of cell cycle

	GO:0006281	DNA repair
	GO:0051301	cell division
	GO:0006310	DNA recombination
	GO:0005819	spindle
	GO:0005815	microtubule organizing center
	GO:0005694	chromosome
	GO:0004003	ATP-dependent DNA helicase activity
	GO:0003887	DNA-directed DNA polymerase activity
	GO:0004386	helicase activity
**microtubules and flagella**	GO:0000226	microtubule cytoskeleton organization
	GO:0007018	microtubule-based movement
	GO:0009296	flagellum assembly
	GO:0030030	cell projection organization
	GO:0042384	cilium assembly
	GO:0015630	microtubule cytoskeleton
	GO:0044430	cytoskeletal part
	GO:0019861	flagellum
	GO:0030286	dynein complex
	GO:0035086	cilium axoneme
	GO:0005813	centrosome
	GO:0005932	microtubule basal body
	GO:0005874	microtubule
	GO:0005929	cilium
	GO:0005856	cytoskeleton
	GO:0005858	axonemal dynein complex
	GO:0035085	cilium axoneme
	GO:0009434	motile cilium
	GO:0030992	intraflagellar transport particle B
	GO:0042995	cell projection
	GO:0044463	cell projection part
	GO:0005876	spindle microtubule
	GO:0003777	microtubule motor activity
	GO:0003774	motor activity
	GO:0004835	tubulin-tyrosine ligase activity
**mitochondria and metabolism**	GO:0006096	glycolysis
	GO:0006122	mitochondrial electron transport, ubiquinol to cytochrome c
	GO:0005983	starch catabolic process
	GO:0006098	pentose-phosphate shunt
	GO:0007005	mitochondrion organization

	GO:0006508	proteolysis
	GO:0015986	ATP synthesis coupled proton transport
	GO:0045261	proton-transporting ATP synthase complex, catalytic core F(1)
	GO:0005750	mitochondrial respiratory chain complex III
	GO:0005747	mitochondrial respiratory chain complex I
	GO:0005739	mitochondrion
	GO:0005759	mitochondrial matrix
	GO:0005741	mitochondrial outer membrane
	GO:0005743	mitochondrial inner membrane
	GO:0046933	proton-transporting ATP synthase activity, rotational mechanism
	GO:0046961	proton-transporting ATPase activity, rotational mechanism
**ribosome and translation**	GO:0006414	translational elongation
	GO:0006412	translation
	GO:0022626	cytosolic ribosome
	GO:0022625	cytosolic large ribosomal subunit
	GO:0022627	cytosolic small ribosomal subunit
	GO:0019843	rRNA binding
	GO:0003735	structural constituent of ribosome
