# Supplementary material for: Prevalence, Evolution, and cis-Regulation of Diel Transcription in Chlamydomonas reinhardtii
Source: G3 (Bethesda). 2014 Oct 28;4(12):2461–71. doi: 10.1534/g3.114.015032 (PMC4267941; doi:10.1534/g3.114.015032)
Supplement: Supporting Information [file supp_g3.114.015032_TableS3.pdf]

Table S3 Optimal parameters and performance measures of SVM classification

Phase	C[1]	R[2]	AUC-ROC	F-measure	Precision	Recall
0	0.01	4	0.64	0.22	0.24	0.20
3	0.1	1.5	0.62	0.21	0.14	0.39
6	0.1	4	0.62	0.19	0.26	0.15
9	0.1	2.5	0.58	0.22	0.27	0.18
12	0.01	3.5	0.64	0.19	0.21	0.18
15	0.1	1.5	0.64	0.27	0.21	0.40
18	0.1	4	0.65	0.23	0.24	0.23
21	0.01	3.5	0.61	0.21	0.38	0.15

1. C = minimum separation

2. R = ratio of negative to positive examples
