# Supplementary material for: Prevalence, Evolution, and cis-Regulation of Diel Transcription in Chlamydomonas reinhardtii
Source: G3 (Bethesda). 2014 Oct 28;4(12):2461–71. doi: 10.1534/g3.114.015032 (PMC4267941; doi:10.1534/g3.114.015032)
Supplement: Supporting Information [file supp_g3.114.015032_TableS4.pdf]

Table S4 "Gold Standard" cycling genes in *C. reinhardtii*

Gene	Name	Reference	Locus	DFT Cyclic Score	COSPOT p-value
ATP2/ARF1	ADP-ribosylation factor	MEMON *et al.* (1995)	Cre17.g698000	0.90	1.9e-02
CAH1	carbonic anhydrase	FUJIWARA *et al.* (1996)	Cre04.g223100	0.76	1.7e-01
CYC4	cytochrome c	JACOBSHAGEN *et al.* (2001)	Cre16.g670950	0.78	6.2e-01
Cytosolic thioredoxin h1	Cytosolic thioredoxin h1	LEMAIRE *et al.* (1999)	Cre09.g391900	0.29	4.0e-01
FBA1	chloroplastic fructose-bisphosphate aldolase	JACOBSHAGEN *et al.* (2001)	Cre01.g006950	0.95	1.2e-02
FBA2	chloroplastic fructose-bisphosphate aldolase	JACOBSHAGEN *et al.* (2001)	Cre02.g093450	0.83	3.4e-02
FBA3	chloroplastic fructose-bisphosphate aldolase	JACOBSHAGEN *et al.* (2001)	Cre05.g234550	0.9	2.3e-02
FBA4	chloroplastic fructose-bisphosphate aldolase	JACOBSHAGEN *et al.* (2001)	Cre02.g115650	0.61	1.9e-02
FNR1	Ferredoxin NADP reductase	LEMAIRE *et al.* (1999)	Cre11.g476750	0.81	2.4e-02
HSP70B	70kd family heat shock protein	JACOBSHAGEN *et al.* (2001)	Cre06.250100	0.32	8.5e-01
LCHII	Chlorophyll binding protein	JACOBSHAGEN *et al.* (1996)	Cre06.g283950	0.82	6.5e-03
PRK1	phosphoribulokinase	LEMAIRE *et al.* (1999)	Cre12.g554800	0.99	1.0e-02
TUB1	Beta-tubulin	JACOBSHAGEN & JOHNSON (1994)	Cre12.g542250	0.67	1.1e-02
TUB2	Beta-tubulin	JACOBSHAGEN & JOHNSON (1994)	Cre12.g549550	0.98	1.1e-02
TufA	Elongation factor Tu	HWANG *et al.* (1996)	Cre06.g259150	0.77	1.1e-02
