# Supplementary material for: Prevalence, Evolution, and cis-Regulation of Diel Transcription in Chlamydomonas reinhardtii
Source: G3 (Bethesda). 2014 Oct 28;4(12):2461–71. doi: 10.1534/g3.114.015032 (PMC4267941; doi:10.1534/g3.114.015032)
Supplement: Supporting Information [file supp_g3.114.015032_TableS5.pdf]

Table S5 Performance COSPOT and DFT on *C. reinhardtii*

Method and α levels	Genome Coverage[1]	Gold Stand Coverage[2]
**COSPOT**		
$\alpha = 0.01$	21.0% (3590)	6.7% (1)
$\alpha = 0.02$	37.4% (6400)	46.7% (7)
$\alpha = 0.05$	54.9% (9392)	73.3% (11)
**DFT**		
$\alpha = 0.01$	29.6% (5061)	33.3% (5)
$\alpha = 0.02$	37.6% (6443)	53.3% (8)
$\alpha = 0.05$	55.8% (9556)	73.3% (11)

1. Parentheses indicated the actual number of genes covered

2. Parentheses indicated how many of 15 gold standard genes are identified as cyclic
