# Supplementary material for: Prevalence, Evolution, and cis-Regulation of Diel Transcription in Chlamydomonas reinhardtii
Source: G3 (Bethesda). 2014 Oct 28;4(12):2461–71. doi: 10.1534/g3.114.015032 (PMC4267941; doi:10.1534/g3.114.015032)
Supplement: Supporting Information [file supp_g3.114.015032_TableS6.pdf]

Table S6 Performance of combining COPSOT and DFT on *C. reinhardtii*

Test P-value	Joint Probability[1]	Overlap[2]	Genome Coverage[3]	Gold Stand Coverage[4]
α = 0.01	0.0134	39.6% (2414)	35.7% (6236)	40.0% (6)
α = 0.02	0.0272	56.7% (4579)	47.2% (8072)	73.3% (11)
α = 0.05	0.0734	73.5% (8024)	61.7% (10552)	86.6% (13)

1. The joint probability of a gene having a score with a p-value of α in either COSPOT or DFT
2. Parentheses indicated the actual number of genes in the overlap set
3. Parentheses indicated the actual number of genes covered
4. Parentheses indicated how many of 15 gold standard genes are identified as cyclic
