# Supplementary material for: Prevalence, Evolution, and cis-Regulation of Diel Transcription in Chlamydomonas reinhardtii
Source: G3 (Bethesda). 2014 Oct 28;4(12):2461–71. doi: 10.1534/g3.114.015032 (PMC4267941; doi:10.1534/g3.114.015032)
Supplement: Supporting Information [file supp_g3.114.015032_FileS1.pdf]

File S1

SUPPLEMENTAL MATERIALS AND METHODS

Determining threshold scores for COSPOT and DFT

MITTAG *et al*. (2005) lists 18 proteins in *C. reinhardtii* which have previously show to exhibit circadian changes in the rate of transcription or concentration of mRNA. Amino acids sequences of these proteins were identified through KEGG and mapped to the *C. reinhardtii* genome using the TBLASTN tool available through Phytozome. We found fifteen proteins which mapped unambiguously to the *C. reinhardtii* genome and had matching annotation, only one of which was not present in the mRNA seq data set (**Table S4**).

To define a cutoff threshold for each of our methods, COSPOT and the DFT, each program was evaluated against our gold-standard set three different *p*-values thresholds (or the equivalent cyclic-score): 0.05, 0.02, and 0.01. For each *p*-value threshold, the coverage of both the gold-standard set and the whole *C. reinhardtii* genome is reported in **Table S5**. At each *p*-value threshold, the union of predictions from methods was used to define cyclically expressed genes in *C. reinhardtii*. As such, the *p*-value of the new two-dimensional threshold is defined by the joint distribution of COSPOT and DFT scores. Calculating this value is complicated by the fact that these scores are highly correlated ($R^2 > 0.7$), but the joint probability can be estimated using a randomized population of expression vectors (**Table S6**). For every test *p*-value threshold, the increase in joint probability (compared to individual significant thresholds) was relatively moderate whereas coverage of the gold standard increased by as much 20% over a single method. We chose to use the combination of COSPOT&DFT as our predictive method with a test *p*-value threshold of 0.02, which balances in the inflation of the joint probability with the coverage of the gold standard set.

The combined method is most effective at excluding non-cycling genes, rather than defining cycling genes, which can be seen by looking at the correlation of both methods at different scoring threshold. (**Figure S7**). While the overall correlation between both methods is high, the correlation amongst highly scoring genes (exceeding the 0.02 threshold for either method) is actually quite low ($R^2 < 0.2$). Genes which score very highly with one method may be at or just below the margin for the other, however, a gene which scores poorly in one method generally scores poorly with the other. Therefore, we chose a more conservative score threshold as a cautionary measure.

Derivation of the model of duplicate gene divergence

Divergence of expression state was modeled using the following system of difference equations:

$$C_{t+1} = C_t \cdot (1-d) + \frac{D_t s}{2} \qquad (3)$$

$$N_{t+1} = N_t \cdot (1-d) + \frac{D_t s}{2} \qquad (4)$$

$$D_{t+1} = D_t \cdot (1-s) + C_t d + N_t d \qquad (5)$$

Where C, N, and D represent the frequencies of cycling, non-cycling, and divergent duplicates at a given *Ks* (subscript "t") and the subsequent *Ks* (subscript "t+1"). The variables d and s are, respectively, the probabilities of divergence from the identical state and reversion to the identical state. Since the null model assumes no bias, d and s are insensitive to whether the identical state is cycling or non-cycling.

Solving equations (3) and (4) for d, we obtain:

$$d = 1 + \frac{D_t s}{2C_t} - \frac{C_{t+1}}{C_t} \qquad (6)$$

$$d = 1 + \frac{D_t s}{2N_t} - \frac{N_{t+1}}{N_t} \qquad (7)$$

Using the property that the right hand sides of (6) and (7) must be equal, we arrive at the following formula for s that depends solely on duplicate frequencies:

$$s = \frac{\frac{2C_{t+1}}{C_t D_t} - \frac{2N_{t+1}}{N_t D_t}}{\frac{1}{C_t} - \frac{1}{N_t}} \qquad (8)$$

Initial conditions were set equal to values of C, D, and N observed at *Ks* = 0.3. We first attempted to fit values for d and s using frequencies at *Ks* 0.3 and 0.6, however because the percentage change in C is greater than N, we obtained a negative value for s. Since s is a probability, this results is unrealistic, so instead we fit d and s using *Ks* 0.6 and 0.9, obtaining values for d (0.42) and s (0.53) that were within [0,1]. Using these parameters, our model was able to replicate the overall behavior we observed, including the initial dip in C, though the percentage change is less than that of N (**Figure S3**). The root mean squared error between our predictions and observation was 0.03.

REFERENCES

MITTAG, M., S. KIAULEHN and C. H. JOHNSON, 2005 The circadian clock in Chlamydomonas reinhardtii. What is it for? What is it similar to? Plant Physiol 137: 399-40
